# Fast Molecular Crystal Structure Prediction Using Sampling by Analogy to Previously Predicted Landscapes

**DOI:** 10.1021/acs.jctc.6c00596

**Published:** 2026-06-09

**Authors:** Jennie Martin, Graeme M. Day

**Affiliations:** School of Chemistry and Chemical Engineering, 7423 University of Southampton, Southampton SO17 1BJ, U.K.

## Abstract

We demonstrate a proof of concept for an approach to fast molecular crystal structure prediction using analogy to hypothetical crystal structures of related molecules. Our method constructs different valid analogues of predicted crystal structures of previously studied molecules and uses these analogues as trial structures in crystal structure prediction workflows for new similar molecules, facilitating successful structure predictions with greatly reduced sampling compared to a conventional CSP search. Early testing on a set of six different families of similar rigid molecules demonstrates that our method can predict both known crystal structures and low lying structures on the target landscape as it would be determined by more complete conventional structure predictions. We show that our approach does so with greater success than quasi-random methods with equally minimal sampling and additionally that trial structures generated via our analogue-based approach lie closer to local minima than structures generated via quasi-random sampling, leading to further reductions in cost. Through these investigations, we show the promise of the proof of concept, which has potential applications where quick structure predictions are needed, such as high throughput studies or preliminary testing.

## Graphical abstract

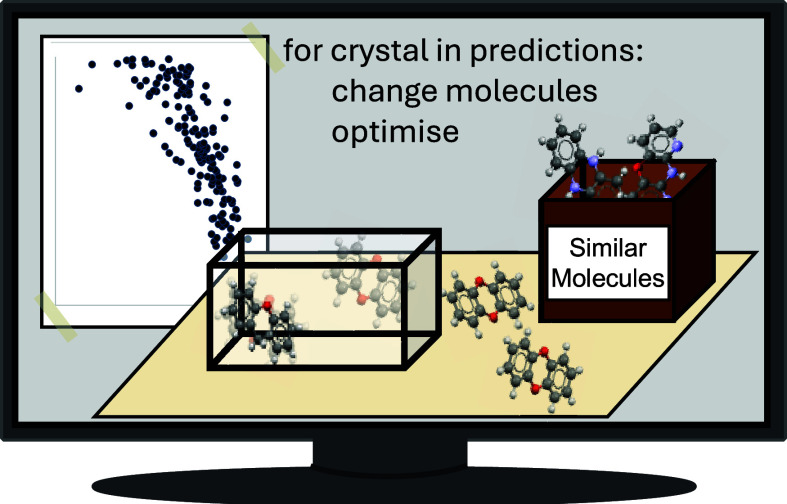

## Introduction

Molecular crystal structure prediction (CSP) is a powerful tool in the field of materials discovery. With rapid growth and development in recent years, CSP methods are now able to predict the experimentally observed crystal structures of a wide range of molecules—with the bank of successful cases covering a variety of chemistries, molecular flexibility and incorporating more complex systems such as salts and cocrystals.^1–8^ This record of success affirms the power of the current approaches in the field. The true promise of CSP methods, however, lies in the prediction of as-yet unknown crystal structures.

As well as being used in conjunction with traditional analytical approaches to aid resolution of crystal structures,^9,10^ CSP methods can also be coupled with property prediction,^11–15^ generating energy-structure–function maps,^16^ which may facilitate the discovery of new functional materials. Important functional properties of a molecular material—such as solubility,^17,18^ compactability,^19^ charge mobility,^20–22^ and porosity^11,12^ can be strongly influenced by the crystal structure. Being able to predict plausible crystal structures of a molecule ahead of synthesis can therefore benefit materials discovery workflows by helping to propose or filter candidate materials and possible crystal structures to target in synthesis. CSP methods have been used in the successful discovery of new useful polymorphs, for example, porous polymorphs.^11,12,23^ Further, CSP is not limited to exploration of a few selected target systems, and has been used in both high throughput searches and within genetic algorithms for exploration of chemical space.^21^ Molecular CSP methods have been shown to be effective, particularly for small organic molecules,^24^ even in spite of commonplace neglect of kinetic factors.

Given the power of CSP-based approaches, steps to reduce the computational and time cost of methods are desirable. CSP methods are often tasked not just with finding a single structure corresponding to the global energetic minimum, but with identifying, as comprehensively as possible, all low-lying local energetic minima—due to the prevalence of polymorphism in molecular crystals. This means that molecular CSP methods usually involve costly and broad sampling, rather than being able to rely upon global optimization processes. Approaches usually revolve around the construction of trial structures to sample structure space, followed by geometry optimization of those trial structures. The optimized trial structures represent local energetic minima and together this set of structures represents the landscape of minima. For simplicity in this work, we refer to this as the “landscape” or “CSP landscape”, despite its lack of information on energetic barriers. A large contributor to the high cost of CSP methods is the extensive sampling required. Due to the high number of degrees of freedom defining molecular crystal structures (including unit cell parameters and positions and orientation of independent molecules), the structure space to explore to find all local minima crystal structures on an energy landscape is vast. This leads to a high computational cost due to a large number of required structure generations and, particularly, subsequent geometry optimizations. The time and resources required for CSP can therefore be reduced by taking steps to limit the necessary exploration of structure space and the number of trialled structures.

If faster, computationally inexpensive CSP methods can be developed—even with some reduction in accuracy and completeness—this would be especially beneficial to particular applications, such as in large-scale exploration of chemical space, where even incomplete exploration of crystal packing can provide valuable guidance,^21^ or for facilitating quick initial testing of new ideas incorporating CSP.

One approach to developing fast CSP methods may be to reduce the number of trialled structures/optimizations required by using prior knowledge to guide sampling of structure space. In essence, it is plausible that if the quality of structure “guesses” can be improved, then fewer such guesses will be required to provide a representative sampling of structural space. An approach that has been used successfully in the prediction of inorganic crystal structures is the use of analogy to other known crystal structures. Structures have been predicted by using element substitution—exchanging the atomic species contained in known structure types to propose new materials.^25,26^

It may also be possible to apply a similar concept to molecular crystal structures—predicting crystal structures based upon the crystal structures of other molecules, which are similar either in shape or in chemistry. A proposed model of crystal packing^27^ compared solid-state molecular packing to the close packing of boxes sharing dimensions related to the molecules. The unit cell dimensions of most known crystal structures lay close to the expected dimensions of one of these limited number of “box packings”—often that that minimized unit cell surface area. This gives hope for analogy-based molecular CSP, suggesting less variation of molecular packings than may be expected, and suggesting that similarly shaped molecules may share similar unit cell dimensions or even molecular packings. Additionally, retrospective analysis has found crystal structure analogues on the CSP landscapes of similar molecules -discovering promising new porous structures via searching for analogues of a known porous material.^12^ However, another study investigated the probability of halogenated pyrolle azaphenacenes crystallizing isostructurally to their nonhalogenated counterparts. Comparison of CSP landscapes of the halogenated molecules to known crystal structures of the nonhalogenated molecules revealed that halogenation tends to disrupt the crystal packing. This suggests that similar molecules may not adopt similar packings, at least in the case of halogenation.^28^ Given these mixed findings, we aim to further investigate the prospect of similarity of crystal structures of similar molecules. Some investigation into analogy-based molecular CSP has been made. For example, one study predicted crystal structures via analogues of experimental structures of similarly shaped molecules, finding the known crystal structures of the target molecules among the predictions in 89% of cases.^29^

However, to our knowledge, the prediction of crystal structures based upon analogues of hypothetical crystal structures, and the evaluation of analogy-based CSP beyond testing known structure recovery, have not been investigated previously. Such investigation was our goal in this paper. Using previously predicted crystal structures, rather than relying upon experimental structures, could help to overcome pitfalls of relying on experimental data—such as limited number of relevant crystal structures, which may prove insufficient. As the molecular CSP field advances, large amounts of predicted structure data are being calculated and shared—meaning that plentiful hypothetical structure data is available. For example, recent studies calculated and shared the CSP landscapes of over 1000 small organic molecules.^24,30^ Analogue-based CSP could extend the use and benefits of hypothetical structure data. Additionally, assessing the ability of analogy-based CSP to predict the wider CSP landscape and compete with other CSP methods should better evaluate the potential of such methods as approximate CSP approaches for a wider and more realistic range of applications.

## Method

### Overview

We developed and tested a proof-of-concept for molecular CSP guided by analogy to previously predicted crystal structures of similar molecules. The approach, which we term “templating CSP”, revolves around extracting templates (crystal structures) from previously predicted landscapes, forming reasonable analogues (equivalent structures albeit comprised of a similar target molecule) and lattice-energy minimizing those structures to derive local energy minima on the CSP landscape of the target molecule.

The method is intended to be accessible to many common CSP workflows—with the templating step representing a complete sampling process—to be paired with any given geometry (re)­optimization steps. Further, the approach is intended to be applicable to a wide array of organic molecules. For the proof of concept, we focused initial exploration on templating using CSP landscapes of intuitively similar molecules. We have developed a templating method that can be used in the prediction of crystal structures of a target molecule provided access to previously predicted CSP landscape(s) of one or more molecules sharing meaningful substructure with the target molecule. Here, meaningful substructure refers to a substructure comprising multiple atoms and sharing similar geometry in 3*D* space. In this work, the approach has been tested on six families of similar molecules—each member of a family either being a smaller/larger analogue of other family members or being interchangeable with all other family members by three or fewer chemical substitutions. These families are shown in Figure 1. Additional constraints during the process ensure the similarity of the structures in 3*D* space. However, one additional tested case (See Templating from Dissimilar Molecules Section) suggested that expansion of the approach to less similar template/target pairs may be worthwhile.

**Figure 1:**
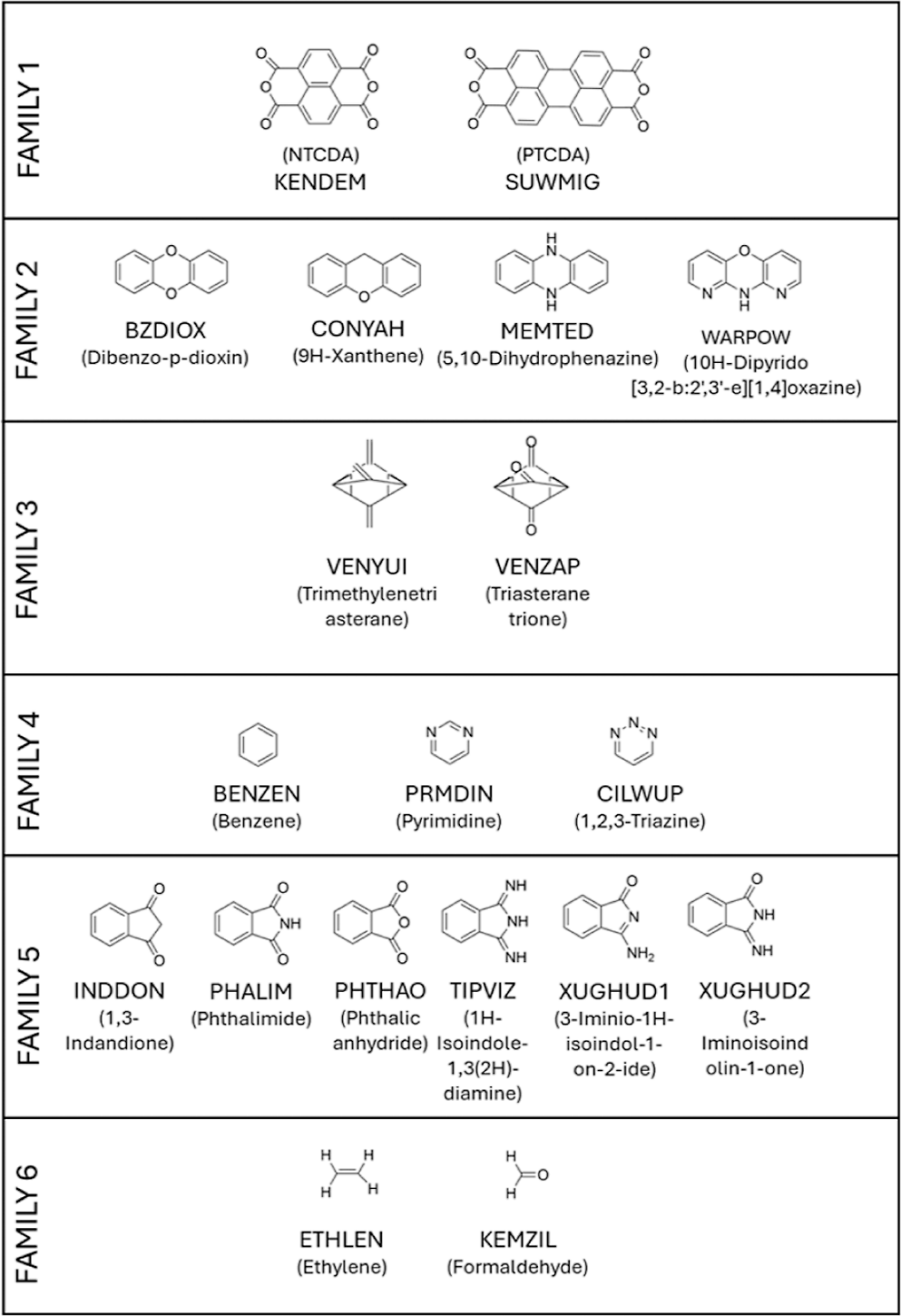
Molecular diagrams of molecules explored in this work, grouped by families of similar molecules. Templating CSP was attempted for all template/target pairings within each family. Molecules are referred to by the alphabetical character string within the CSD^31^ REFCODES of their known structures. Compound names, or more common names are given in brackets and may be used in the paper. The 1,2 numbering of tautomeric pair XUGHUD1 and XUGHUD2, is chosen arbitrarily to distinguish the conformers and does not relate to numbering within the CSD or the REFCODES of their respective known crystals.

### Analogue Construction

The approach uses as templates previously predicted crystal structures of similar molecules—which have been extracted from CSP data sets. Rather than using merely the global minimum energy structure, multiple templates from each original landscape are used. Depending on costs and requirements, this may be all structures within the available landscape or a subset of structures—thus far we have explored use of the full landscapes as well as selection of templates that fall within given energy windows (25 and 15 kJ/mol) relative to the global energy minimum.

The method then attempts to construct all “reasonable analogues” of each given template. The concept of what represents a reasonable analogue for a given crystal structure is not a given, and there are aspects of the construction that could be debated. However, as a minimum, analogous molecular crystals should share similar packing of the molecules. Our method seeks to replicate the unit cell parameters and the orientations and positions of molecules relative to the unit cell axes. This is achieved by first reasonably replicating the position and orientation of asymmetric unit molecules by overlaying the original and target molecules.

The molecules are overlaid so as to best overlay their maximum common substructure. This method was chosen as it aligns the shared chemical and shape-based character of the molecules within the respective molecular packings. Consideration of any symmetry of the substructure and/or multiple instances of substructure within the molecule(s) further allows for the enumeration of all valid analogues of each template. For each template, we form separate analogues for each pair of instances of the substructure in the template/target molecules, in which initial positioning and orientation of the analogue asymmetric unit molecules is performed by overlaying the corresponding substructure instances. Furthermore, if the substructure itself is symmetric, there can be multiple equally valid ways of overlaying a given pair of substructure instances. For example a pair of substructure instances could be overlaid both before and after rotation of one instance of the substructure along an axis with rotational symmetry—if one or both of the full molecules themselves would not be equivalent under that transformation, then this may lead to different analogues. We call these multiple overlays of a single pair of substructure instances “isomorphic overlays” and consider all such cases. These considerations are necessary to avoid arbitrary restriction and construct all valid analogues. Nonexhaustive examples of the construction of multiple analogues differing by substructure instance or isomorphic overlay are given in Figure 2. Additional discussion of the considerations for constructing all valid analogues is given in Section S4. The maximum common substructure of original and target molecules, and the instances of that substructure are identified using the Python Application Programming Interface provided by the Cambridge Structural Database Team (CSD API).^32^ The search is restricted to ensure that the substructure must be connected—but bond typing is not considered. Isomorphic ways in which to overlay the substructure instances are identified using molecular graphs. All potential overlays are filtered to ensure meaningful interpretation in physical space (See Limitations Section).

**Figure 2:**
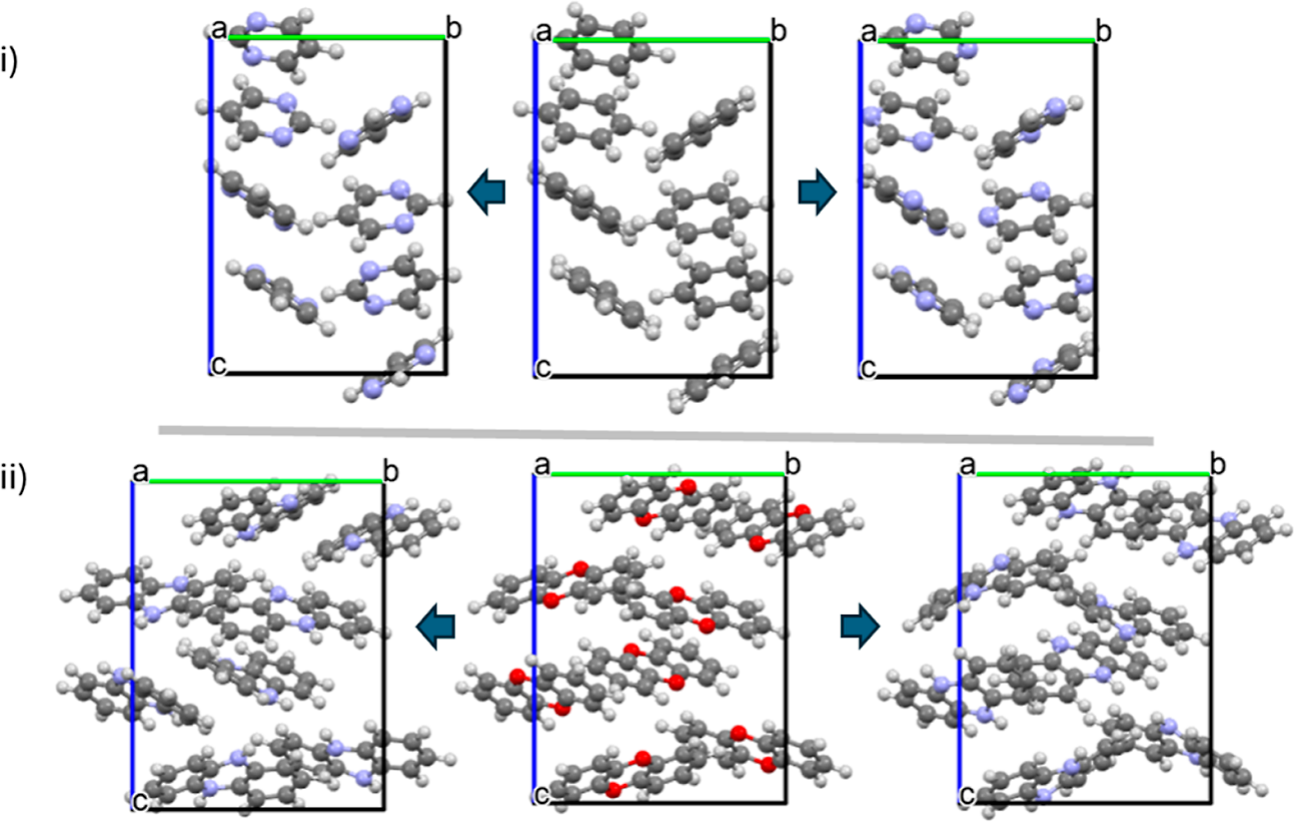
Examples of multiple valid analogues (left and right) formed from a single template (center). Examples include analogues differing by the substructure instances used to determine molecular overlay in their construction (i) and analogues differing by different isomorphic overlays of a given pair of substructure instances (ii). In the instance of BENZEN/PRMDIN as a template/target pair (as in (i)) there are six instances of the maximum common substructure to consider. For brevity, only two are shown here. In the case of BZDIOX/MEMTED as a template target pair (ii), the substructure was symmetric—meaning that the molecules could be overlaid in two different ways (differing by transformation of a substructure instance under a rotation). This results in different analogues due to nonplanarity of the MEMTED target molecule.

Implementation of overlays utilizes the indexing of individual atoms within molecule/crystal objects. Consistent indexing can be enforced via preprocessing—setting indexing to match a desired reference.

After the initial positioning and orienting of the new molecules, their positions are further shifted such that the positions of their geometric centroid relative to the unit cell axes match those of the template asymmetric unit cell molecules. We viewed this as better representing the conceptual analogue and such shifting may better replicate the molecular packing, particularly where the shared substructure of the original and target molecules is not centered within one or both of the molecules.

The remaining unit cell molecules are then positioned and oriented by application of the template space group operators. This results in formation of crystal structures that are visibly analogous to the templates.

In some instances, particularly where the new molecule is larger along any given dimension than the original molecule, this analogue formation can result in clashes between the molecules—in which intermolecular atomic separations are unphysically short. Our method detects such clashes using a combination of molecular graphs and interatomic distance measurements and attempts to resolve them by enlarging the unit cell. When a clash is detected, the shortest unit cell length is increased by 1 Å and all molecules are shifted so as to maintain the fractional coordinates of their centroids. This process is repeated until no further clashes are detected or until reaching an excessive number of enlargements (defined for the proof of concept as 60 enlargements)—at which point the analogue is rejected.

Lastly, all constructed analogues are filtered via comparison of simulated pXRD patterns to remove duplicate structures, removing the potential of needless geometry optimizations.

### Limitations

The construction of analogues via substructure overlay can lead to some restrictions in the applicability of the method. Namely, the template and target molecules must share meaningful substructure, i.e of multiple atoms so as to suitably guide overlay. Additionally, the identified substructure instances must be physically overlayable in 3*D* space with minimal error. Substructure instances in our workflow were identified via MaximumCommonSubstructure and SubstructureSearch functionalities in the CSD API^32^—identifying substructure based solely on connectivity, without knowledge of 3D geometry. We excluded unreasonable overlays identified by these functionalities, or by the enumeration of isomorphic overlays, by implementation of a limit ≤2 Å RMSD in atomic positions of the substructure overlay for inclusion of the analogue in predictions. Fuller discussion of these limitations is provided in Section S4.

Additional problems may be faced when using template/target pairs for which the target landscape is significantly more populous than the template landscape. As a single analogue can only lead to one minimum on the target landscape, such cases may encounter an inherent limit on their predictive power. Extensions of the approach to address this could incorporate the pooling of templates from multiple landscapes or the implementation of additional sampling, such as with basin hopping,^33–36^ to explore the landscape around the minima predicted by templating.

### Workflow Summary

Our proposed workflow for templating CSP would be to generate trial structures via formation of analogues of a suitable selection of templates, followed by geometry optimization of those trial structures to obtain local minima on the energy landscape. The full workflow is summarized in Figure 3.

**Figure 3:**
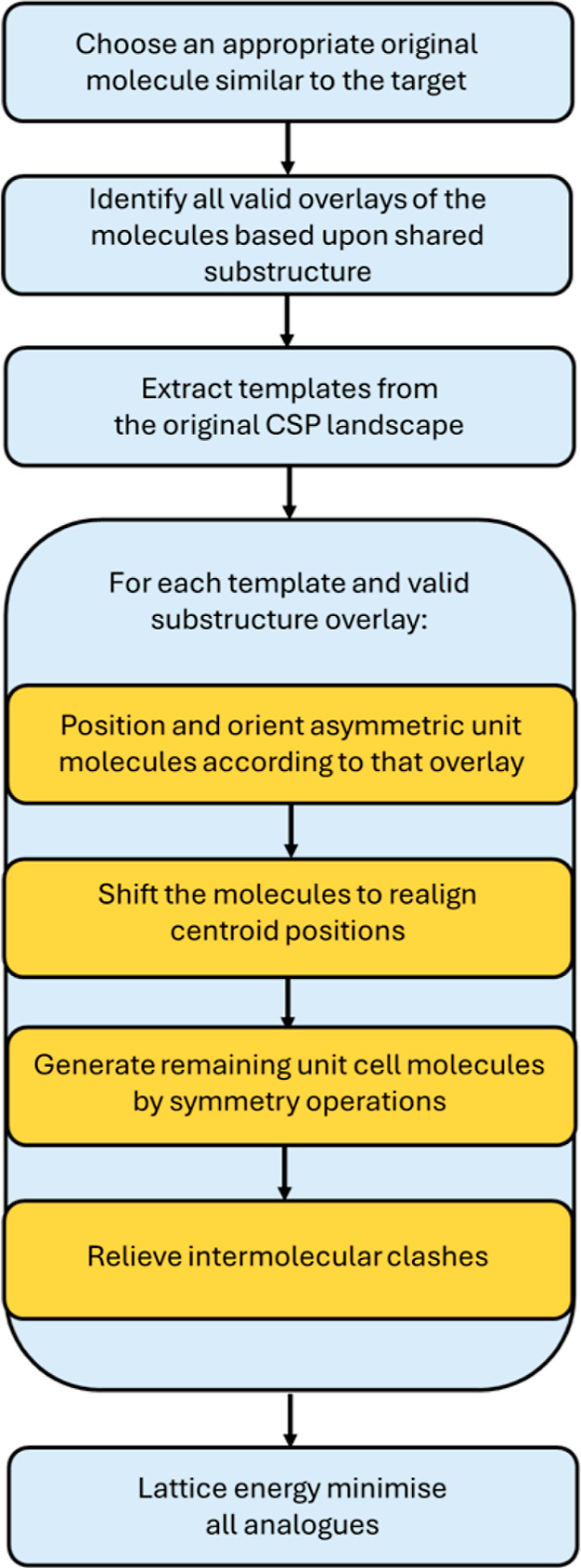
Flowchart summarizing the full templating CSP workflow.

In this work, to facilitate comparisons to quasi-random CSP, we imitate the optimization workflow used in the global lattice energy explorer (GLEE) approach,^37^ in which all crystal structures are ultimately lattice-energy minimized using a pairwise interatomic force field combined with permanent electrostatic contributions from distributed atomic multipoles up to the hexadecapole. All cases in this work used the FIT^38,39^ force field and multipoles calculated using DFT with either B3LYP^40–42^ or PBE0 ^43,44^ functionals; these were selected so as to match those multipoles used in calculation of the target landscapes (see below). The molecular conformations used in the construction of analogues were also those used in calculation of the target landscapes. Figure 4 shows examples of template crystal structures, and one of their corresponding analogues both before and after geometry optimization of the analogue. The final “Optimized Analogues” in the figure are examples of final structures that would be found on the relevant “Templating CSP” landscapes.

**Figure 4:**
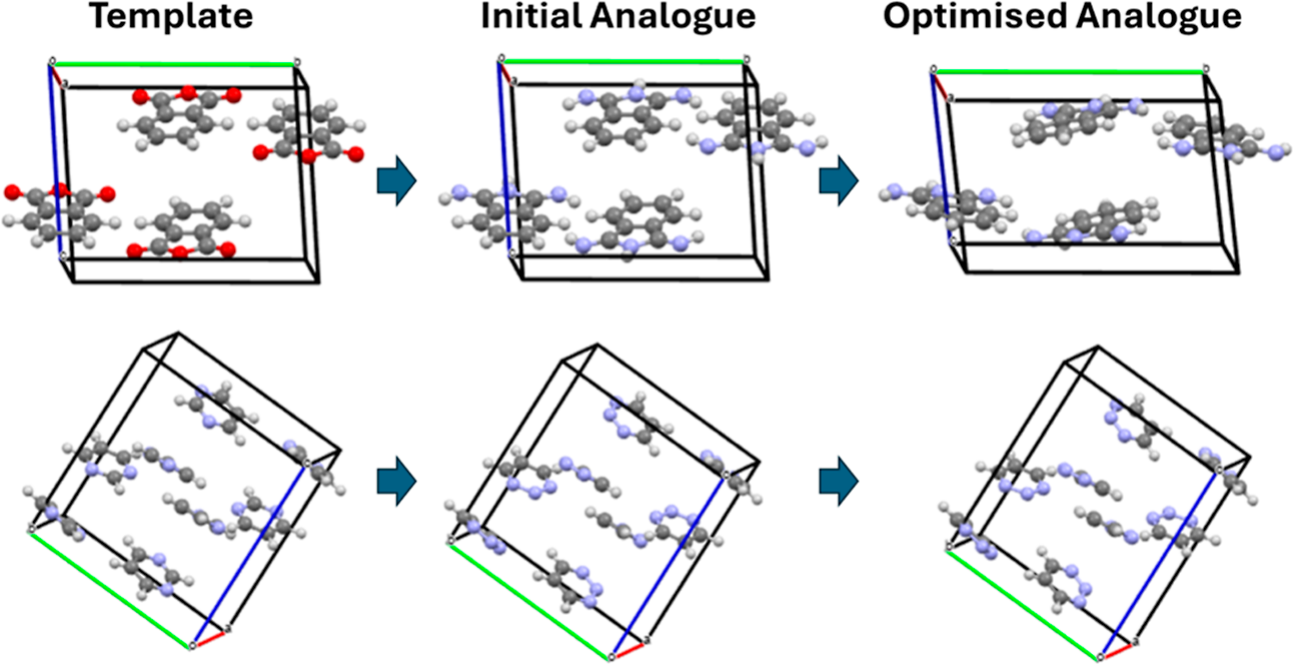
Examples of template crystal structures, the initial analogues formed from them, and the final optimized analogue—i.e the resulting structure on the templating CSP landscape. Cases shown are for PHTHAO/TIPVIZ and PRMDIN/CILWUP template/target pairs.

Lastly, the set of successfully optimized analogues is filtered via comparison of simulated pXRD patterns^45,46^ to remove duplicates.

To investigate the role of template selection, we trialled selection of templates from original landscapes by application of different relative lattice energy windows: full landscape, 25 kJ/mol window, 15 kJ/mol window. We then obtained the corresponding templating CSP landscapes, by creating analogues for all crystal structures within these energy windows on the original landscape.

## Results and Discussion

### Evaluation Criteria

To assess the performance of the templating approach, we compared the method against the benchmark of quasi-random CSP. (Quasi) random approaches to CSP are commonly used, and have proved effective in predicting known molecular crystal structures, for example in the most recent CSP blind test.^7,8^ In particular, the GLEE protocol^37^ used for QR CSP in this work has performed well^7,8^ and has recently been tested upon a set of over 1000 small molecules, successfully predicting all but 6 of the corresponding known *Z*′ ≤ 1 crystal structures.^24^

As performance metrics, we explored the recovery of known experimental structures (RK) and recovery percentage (RP) of low energy structures that were predicted by a conventional quasi-random CSP workflow. Recovery here means that the prediction set obtained contained a structure closely matching the target structure, be that target an experimentally known structure or a conventionally predicted structure.

Due to the small number of known polymorphs per system for the cases explored in this work—a maximum of two polymorphs per system—recovery of known structures (RK) is described simply by “*x* of *y*”, where *y* is the number of known polymorphs and *x* is the number of polymorphs recovered by the CSP.

Recovery percentage (RP) is the percentage of unique low energy minima on the target CSP landscape (quasi-random CSP is used to generate a target reference) that were recovered by templating CSP

1 $$RP=\frac{No.unique\;low\;energy\;traditional\;minima\;recovered}{No.\;unique\;low\;energy\;minima\;in\;traditional\;CSP}\times 100$$

To ensure thorough assessment of the method, we assessed the performance metrics conservatively, using the COMPACK^47^ method as implemented in the CSD API^32^ for structure comparisons. Target structures were only declared to be recovered if there existed at least one templating CSP structure for which 30/30 molecules of representative 30 molecule clusters of the respective crystal structures can be overlaid to within tolerances of 30% for interatomic distances and 30° for angles (assessing RK) or 20% and 20° (for assessing RP). Due to potential imprecision in experimental crystal solution and distance of the structure from a true minimum on the structural landscape at the level of theory used, for recovery of known structures all such 30/30 overlays were deemed to be indicative of recovery. However, when assessing RP, a target structure was only deemed to be recovered if the RMSD in atomic positions of the corresponding overlay was less than or equal to 0.05 Å. This strict criterion ensures certainty of recovery. Figure 5 shows examples of results demonstrating the required quality of overlays. Figure 5a shows a known structure recovery, as there is no cutoff for quality set for experimental matches in this work, the example is chosen to demonstrate quality similar to the average quality seen in our results. Figure 5b shows a low-energy target structure recovery with RMSD close to the 0.05 Å cutoff.

**Figure 5:**
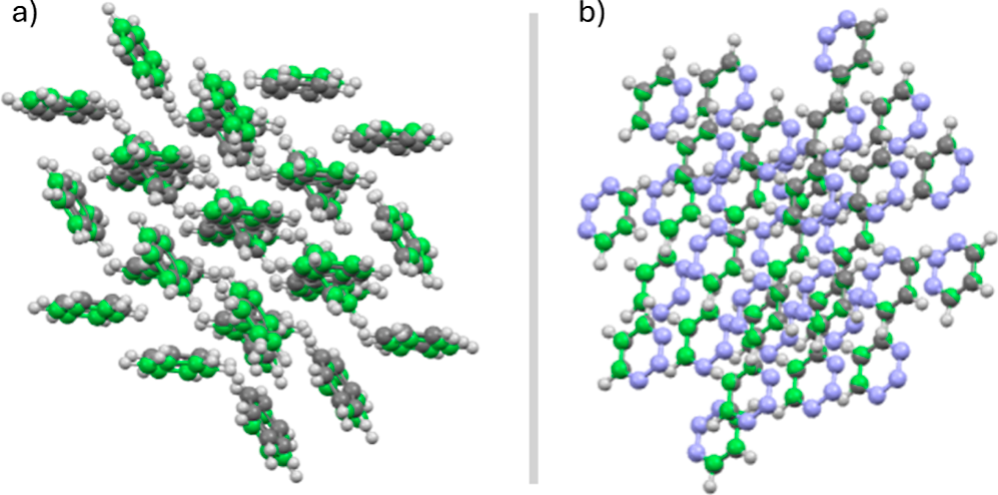
Examples of an overlay between structure predicted by templating CSP (green) and a known structure (a) or low-energy target structure (b) (Element-Color).(a) shows an overlay between a structure of BENZEN predicted by templating CSP and known structure BENZEN03, the RMSD of the overlay is 0.415 Å, close to the average match-quality in this work. (b) shows an overlay between a structure of CILWUP predicted by templating CSP and one predicted by traditional QR CSP. The overlay has RMSD_30_ = 0.041 Å.

These criteria were chosen to appropriately measure the most important aspects of the method. Recovery of known crystal structures is a clear goal when testing any CSP method, as such structures are the only certain examples of structures that can be crystallized in reality. The recovery of other low energy crystal structures predicted by the benchmark QR approach is important as this is the region of the landscape from which structures are most likely to be found experimentally. Prediction of these structures is important as these structures have the potential to represent possible, though currently unobserved, polymorphs. Additionally, prediction of structures in this region may be used for guiding crystallization attempts in materials discovery, particularly if a structure in that region has promising properties. In this work we focused upon recovery of structures in the lowest 7.5 kJ/mol, a sensible window justified by the finding that 95% of all known polymorphs are separated in energy by less than 7.2 kJ/mol.^48^

Quasi-random CSP to obtain the target landscapes for benchmarking was performed using the GLEE protocol,^37^ implemented in molCSPy^49^—principally relying upon QR generation of trial structures that are then lattice energy minimized within the DMACRYS software^50^ using an interatomic force field (FIT) with electrostatic contributions from distributed atomic multipoles (FIT + DMA). Sampling was performed so as to obtain 10,000 structures with one molecule in the asymmetric unit in each of the 10 or 26 most common space groups for organic crystals in the CSD,^31^ before removal of duplicates using comparison of simulated PXRD patterns and geometric overlay comparisons (see ref 28 and Section S1). The FIT + DMA optimization is known to be effective for crystal structures of small molecules, having been tested on the X23 ^51^ benchmark set.^52^ With the exception of systems NTCDA, PTCDA and XUGHUD2 (see Figure 1), all target landscapes in this work were taken from a recent CSP study of over 1000 small molecules.^24^ Target landscapes for NTCDA, PTCDA and the tautomer XUGHUD2 were calculated for this work (See Section S1). These predicted landscapes serve a dual purpose. The landscape of a given molecule *A* provides the target landscapes for templating CSP runs where *A* is the target molecule, and provides the source of templates for analogue formation where *A* is the template molecule.

To ensure that the target landscapes were sufficiently comprehensively sampled, we investigated all template/target pairings and the respective templating CSP landscapes formed when using all available crystal structures of the template molecule. We compared these landscapes (after thorough duplicate removal via geometric overlays) and counted low-energy structures predicted by templating CSP that were not present on the target landscape. We found such cases to be negligibly rare (See Section S2). Therefore, we can view the templating predictions as a subset of the full target landscape and evaluate the success of the approach appropriately.

Recall that we trialled templating CSP for all target template/pairs within several identified families of similar molecules (Figure 1). These families include a manually selected pair of differently sized analogues (NTCDA/PTCDA) as well as several families of molecules from the large collection of over 1000 CSP landscapes.^24^ Possible families of molecules which can be interchanged by 3 or fewer chemical substitutions along the molecule backbone were identified automatically, via identification of interchangeable pairs using character-based comparisons of SMILES strings. Chemical substitutions were restricted to be single heavy-atom swaps, but hydrogen positions were not tracked—facilitating, for example, the replacement of a CH with a nitrogen atom. The replacement of hydrogens with heavy atoms—such as in a H/F swap, was not treated as a permissible substitution. Identification of molecule pairs was followed by identification of groupings using the Reverse Cuthill-McKee functionality^53^ in SciPy^54^ (For further methodological details see Section S3). The resulting set of possible families was manually filtered to select families covering a range of molecular geometries and functional groups.

In this work we refer to templating CSP runs using the language “*X* in *Y*”, where *X* is the target molecule for which predictions are being performed, and *Y* is the template molecule, previously predicted crystal structures of which are used as templates in analogue formation.

### Evaluation

#### Recovery of Known Structures

We first turn to the assessment of recovery of known structures. Table 1 shows the overall recovery of known structures, and the mean quality of the corresponding structure matches as a function of the starting set—i.e the selection window used to extract template structures from the original landscape.

**Table 1:** Table of Performance, across Trialled Systems, with Regard to the Recovery of Known Crystal Structures via Templating CSP with Different Template Selection Windows^a^

starting set	% successful pairings	% successful targets	mean match RMSD_30_ (Å)
full landscape	72	84	0.421
25 kJ/mol	72	84	0.446
15 kJ/mol	59	84	0.480
small QR	48	68	0.420

a Metrics shown are the percentage of template/target molecule pairings for which all desired known crystal structures could be predicted via templating CSP for that pairing, the percentage of targets for which all desired known crystal structures could be predicted via templating CSP using at least one of the trialled template molecules, and the mean RMSD_30_ of matches to the known crystal structures. This mean is calculated by first finding the lowest RMSD match to each known polymorph from the CSPs for each template/target molecule pairing, and averaging across all those results. Cases where a match was not found were excluded from the calculation. Shown in grey are the same performance metrics assessed for quasi-random CSP with limited sampling (small QR). For small QR, we conducted separate quasi-random CSP corresponding to each template/target molecule pair—requesting the same number of sample structures in each spacegroup as were produced unique initial analogues in that space group during templating CSP for that molecule pair using the 25 kJ/mol template selection window.

For 72% of trialled template/target molecule pairings, all high-quality known *Z*′ ≤ 1 polymorphs (see Section S5) of the target could be predicted when using all available templates during analogue formation (i.e when forming analogues of the full template molecule landscape). More promisingly, for 84% of targets, there existed a trialled template molecule for which, when performing templating CSP by forming analogues of all available crystal structures of that template molecule, all high-quality known *Z*′ ≤ 1 polymorphs of the target could be predicted. The three targets for which this is not the case are XUGHUD1, PRMDIN, and KEMZIL. KEMZIL (formaldehyde), which has only one known polymorph meeting the requirement, is a difficult case, as the change in structure from ethylene is a relatively large perturbation for such a small molecule. XUGHUD1 and PRMDIN both have two polymorphs. One polymorph of XUGHUD1 and one polymorph of PRMDIN were predicted via templating CSP, representing partial recovery for these cases. The quality of successful recovery was generally reasonable (Table 1), with the mean RMSD between the known structure, and the best predicted structure (from each successful template/target pairing CSP) recovering it being 0.421 Å.

When sampling is reduced, and analogues were formed only of templates lying within 25 kJ/mol of the global minimum on their respective landscapes, these statistics were matched (Table 1). The mean match quality with this reduced sampling was 0.446 Å. Performance when using only templates lying in a 15 kJ/mol window was weaker. While still recovering all known structures for 84% of targets when using as templates the crystal structures of some template molecule, recovery was only achieved in 59% of trialled cases (template/target pairings) and with further reduced mean match quality, suggesting that such minimal CSP could be less powerful. (See Section S6 for full details on recovery of known structures.).

While the performance, particularly the percentage of successful pairings, is not on par with traditional methods, this is to be expected. The sampling used is very minimal compared to the sampling that would be employed for confidence in a traditional CSP search. The extent of sampling in templating CSP here varies, as it is dependent upon the number of available templates. The number of unique analogues formed prior to minimization in this work ranges from just 18 to 24,996, with the average number of unique analogues formed when using all available templates in a 25 kJ/mol window being 1893. In contrast, the QR CSP used to obtain the target landscapes sampled from 100,000 to 260,000 successfully optimized trial structures. A useful evaluation here is to compare the performance of templating CSP to what would have been achieved via quasi-random methods had the sampling been equally minimal. We performed small quasi random structure searches to compare to the templating results for the 25 kJ/mol windows. We view the 25 kJ/mol window as being ideal for these comparisons as not only does it represent the middle-ground of work on templating CSP, but the results for the 25 kJ/mol template selection set shown throughout this work demonstrate that it can achieve adequate performance close to that of use of a full landscape of templates, without excessive sampling and cost.

For each template—target pair, we performed a QR CSP search requesting the same number of structures in each space group as existed unique initial analogues in that space group in the corresponding templating CSP. Then, we evaluated the recovery of known structures—as when evaluating templating CSP (Table 1). The quality of recovery was similar, with an average RMSD of 0.420 Å. However, recovering all known structures was only possible for 48% of template/target pairings, and for 68% of targets. This demonstrates the potential of templating CSP to outperform QR CSP in cases requiring a protocol with highly restricted sampling. This suggests that the success of templating CSP in recovering known structures is not merely due to chance, and that the guided nature of the sampling is impactful.

Additionally, it is important to remember that in a single attempted templating CSP run (i.e for one template/target pairing and template selection window), all templates are being taken from the crystal structures of just one other molecule. It is not unreasonable that in some cases, the experimental crystal structure of one molecule simply does not correspond to any local minima on the CSP landscape of the other molecule. However, the more promising figure of 84% for targets having their known structures be predictable by some case of templating offers reassurance for the method. This indicates that in most cases, there exists some similar molecule for which some CSP landscape minima are analogous to the known structures of the target molecule. And, as the number of possible template molecules explored for each target is small (1–5), such a suitable template molecule may not be rare in a suitable class of related molecules. Therefore, when the approach is implemented beyond the proof of concept, it may benefit from generation of analogues of a “pool” of templates from multiple systems. Additional research into identification of trends for identifying effective template molecules for a given target may also be warranted.

#### Recovery Percentage of Low-Energy Target Structures

Assessment of recovery of low energy target structures reflected well on the potential of templating CSP. Table 2 shows the mean, across trialled template/target pairs, of the percentage recovery of the lowest 7.5 kJ/mol of the target landscapes when using templating CSP with different template selection windows. Full results, broken down by template/target pair, can be found in Section S7.

**Table 2:** Table of Mean Values, across Trialled Template/Target Molecule Pairings, of the Percentage Recovery of Low-Energy Target Structures from Templating CSP with the Corresponding Molecule Pairings for Different Template Selection Windows Shown in Grey are the Mean Percentage Recovery for Quasi-Random CSP with Limited Sampling (Small QR)^a^

starting set	mean % recovery of low-energy target structures
full landscape	74.9
25 kJ/mol	70.6
15 kJ/mol	52.8
small QR	68.4

a For small QR, we conducted separate quasi-random CSP corresponding to each template/target molecule pair—requesting the same number of sample structures in each space group as were produced unique initial analogues in that space group during templating CSP for that molecule pair using the 25 kJ/mol template selection window.

Templating CSP runs recovered on average 74.9% or 70.6% of structures in the lowest 7.5 kJ/mol region of the target landscape when using a full landscape of templates or using all templates within 25 kJ/mol, respectively. Performance drops, however, when using only templates in a 15 kJ/mol window on the respective landscape, with an average of just 52.8% recovery. Similarly to recovery of known structures, this represents a drop in performance of templating CSP when using such a restricted template set, suggesting that templating CSP with this minimal sampling may prove insufficient.

Performance does also vary across template–target pairs, as demonstrated in Figure 6, which shows a histogram of recovery percentages across all attempted templating CSP runs. Figure 7 provide a more detailed picture—broken down by molecule family—showing the recovery percentages for each template/target pair, separated by family, when using all available templates within a 25 kJ/mol window. (Equivalent plots for other template selection windows can be seen in Section S8). The plots separated by family demonstrate that there is some variation in performance for different families of molecules. For instance, both the spread and the average of RP values vary between families. It may be that there are certain shared molecular characteristics that make a family of molecules better candidates for templating. For example, one might expect that families of molecules differing by size (Family 1) would demonstrate different performance to those differing by functional groups (Families 2–6)—due to possible differences in the relative importance of steric factors and electrostatic interactions. While most molecule families investigated here contain molecules sharing the same dimensionality, Family 2 combines planar molecules with molecules that deviate from perfect planarity. Naively, it could be thought that this could explain the large variation in templating performance across Family 2. However, the same spread is seen for Family 5, which features only planar molecules. Importantly for this discussion, the results also show notable variation across template/target parings within families. As a result of this finding, it is harder to gain meaningful insight into the physical factors that may lead to greater success from one molecule family over another, as these interfamily performance variations must be disentangled from the intrafamily variations. Further study, across a wider set of molecules/families could provide additional data points to facilitate these investigations.

**Figure 6:**
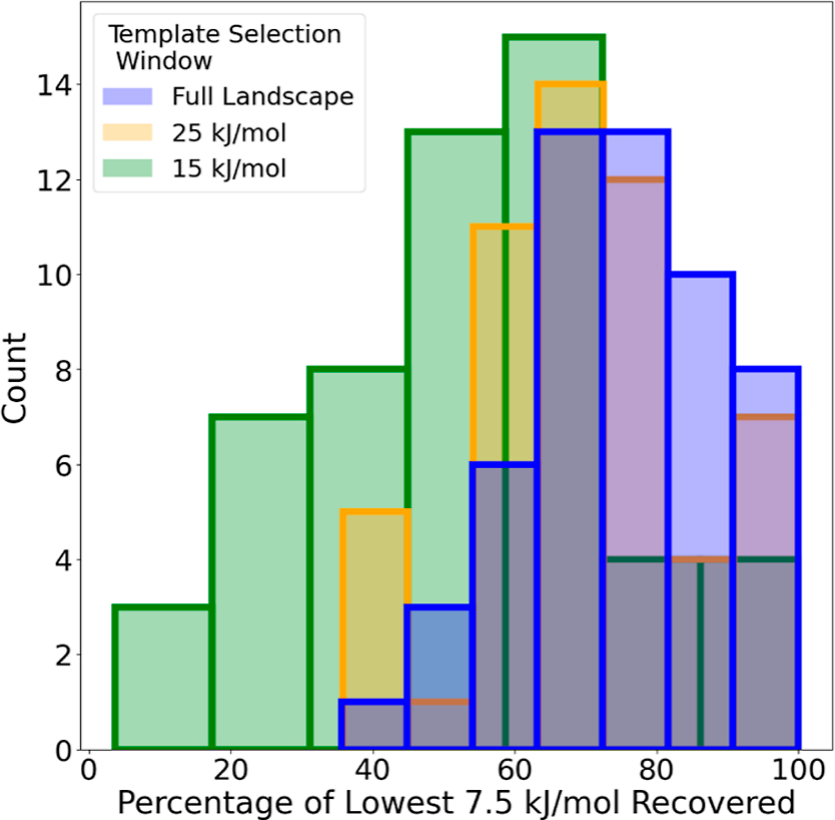
Histograms showing the frequency of different recovery percentages achieved across all templating CSP runs. Each template selection window was analyzed separately, denoted by color. Binning was performed automatically for each histogram, as the differing ranges/distributions were considered informative.

**Figure 7:**
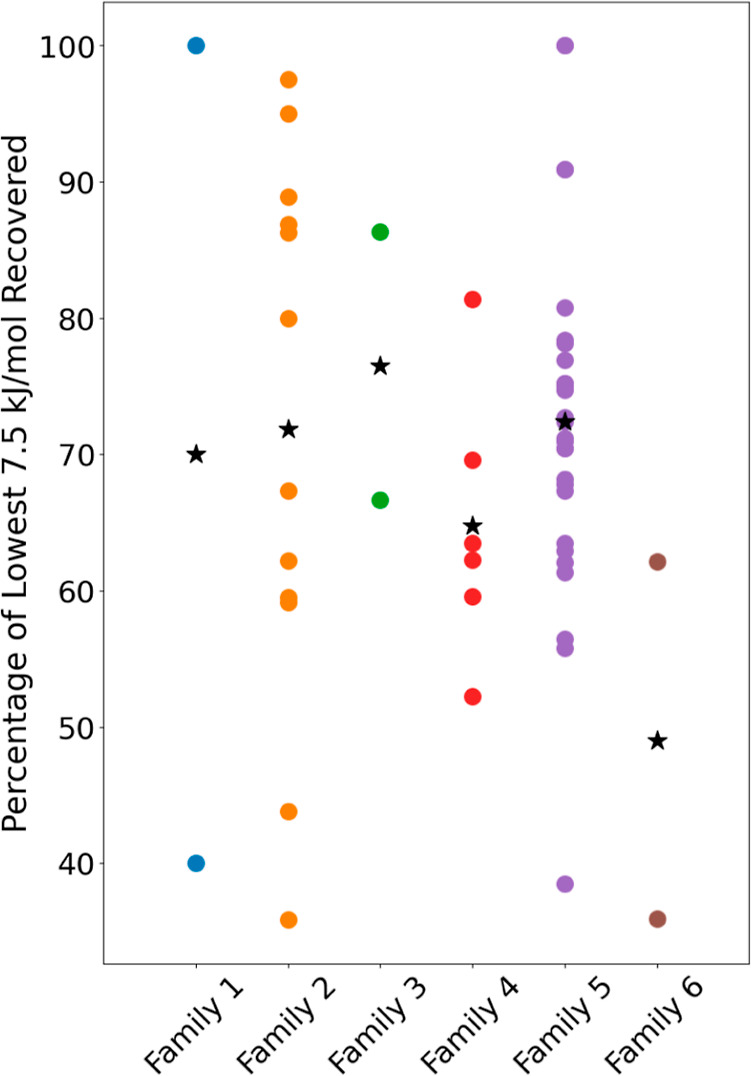
Plot showing the recovery percentage values for templating CSP runs, using a 25 kJ/mol template selection window. Results are grouped by the family of similar molecules (See Figure 1) to which the template/target pair belongs. Black stars represent the average, across temple/target pairs for that family.

In studies beyond the proof of concept, further investigation should be made to garner explainable insight into the connection between the choice of template/target pairs or families and the predictive power of templating CSP. Such investigations could call upon further testing of size inconsistent template/target pairs to more deeply investigate the potential of such families and explore comparison of molecular similarity measures to templating performance. Other investigation in this area could explore determination of the fragments within a molecule under investigation that are most likely to influence lattice energy/packing. This may use tools such as Local Energy Decomposition (available in ORCA^55,56^ ) or understanding of likely energetic contributions from different functional groups, which, for example, have been derived previously using machine-learning.^57^ These findings could then be related to the performance of templating for template/target pairings in which these fragments are altered/maintained.

Given the impact of the template selection window on performance, it is useful to investigate how much templating CSP performance is impacted simply by the extent of the sampling. A simple test of this can be seen in Figure 8, which plots the recovery percentage for each templating CSP run against the ratio between the number of analogues formed and the number of target structures to predict. This serves as a measure of difficulty of the task when viewed purely as a “numbers game”.

**Figure 8:**
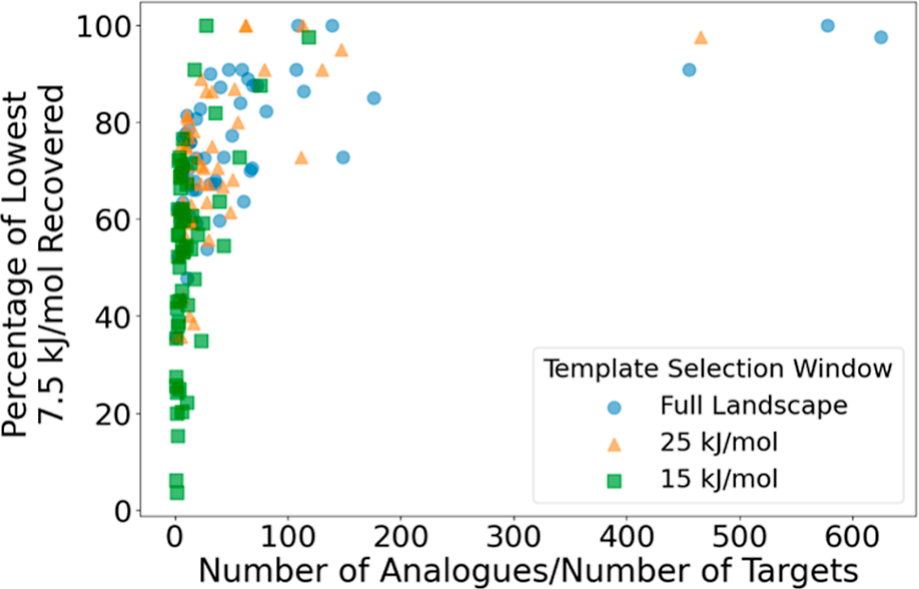
Scatter plot indicting the relationship between the recovery percentage and the “statistical difficulty” (analogues/targets ratio) for each templating/target pair explored. Color indicates the template selection window used.

The plot indicates a relationship in which template–target pairs which, from a purely statistical perspective, present a more difficult challenge for templating CSP tend, unsurprisingly, to lead to poorer results. It appears that, overall, cases with template/target pairs with higher target/analogue structure ratios recovered fewer of the low-energy target landscape structures. However, this is not universal. To explore the extent of this trend, we calculated the Kendall rank correlation coefficients between the target/analogue structure ratios and the percentage recovery of the lowest 7.5 kJ/mol of the target landscape for each starting set. These tests indicated a reasonable—though not complete-correlation (τ = 0.410–0.482).

The moderate strength of this correlation indicates that while the “statistical difficulty” of a templating task and the performance of templating CSP for that task are correlated, this is not definitive. The scatter away from this relationship indicates either significant variation in performance allowed due to chance or the presence of other factors impacting the success of templating for a given target/templating pair.

Given this incomplete correlation, it appears that the recovery performance is not determined entirely by the extent of the sampling. Therefore, it cannot be assumed that alternative methods of trial structure construction, using an equivalent number of samples, will yield structure prediction with equal performance.

To investigate the potential of templating CSP relative to traditional methods, we evaluated the performance of templating to the performance of quasi-random CSP methods when using equally small sampling. Recall that traditional quasi random CSP oversamples by design in order to be as complete as possible, and so it is not fair to assume that good percentage recovery by templating methods with small sampling inherently shows the method’s superiority over QR. QR CSP with small sampling will also recover some percentage of the crucial low energy region of the target landscapes. To fairly evaluate the impact of templating in guiding the sampling of structure space, the key results should be compared to those that would be achieved by a similarly restrictive quasi-random structure search. To investigate this, we returned to the small quasi random structure searches performed for comparison to the templating results when using structures extracted from the 25 kJ/mol windows. For these small QR searches, we evaluated the recovery of low energy structures from the target landscapes—as when evaluating templating CSP.

Figure 9a shows the percentage recovery via templating CSP and small QR CSP for each case. Here, points above the line *x* = *y* represent cases where templating CSP outperforms QR. Figure 9b is a histogram of the differences in performance between templating and QR CSP.

**Figure 9:**
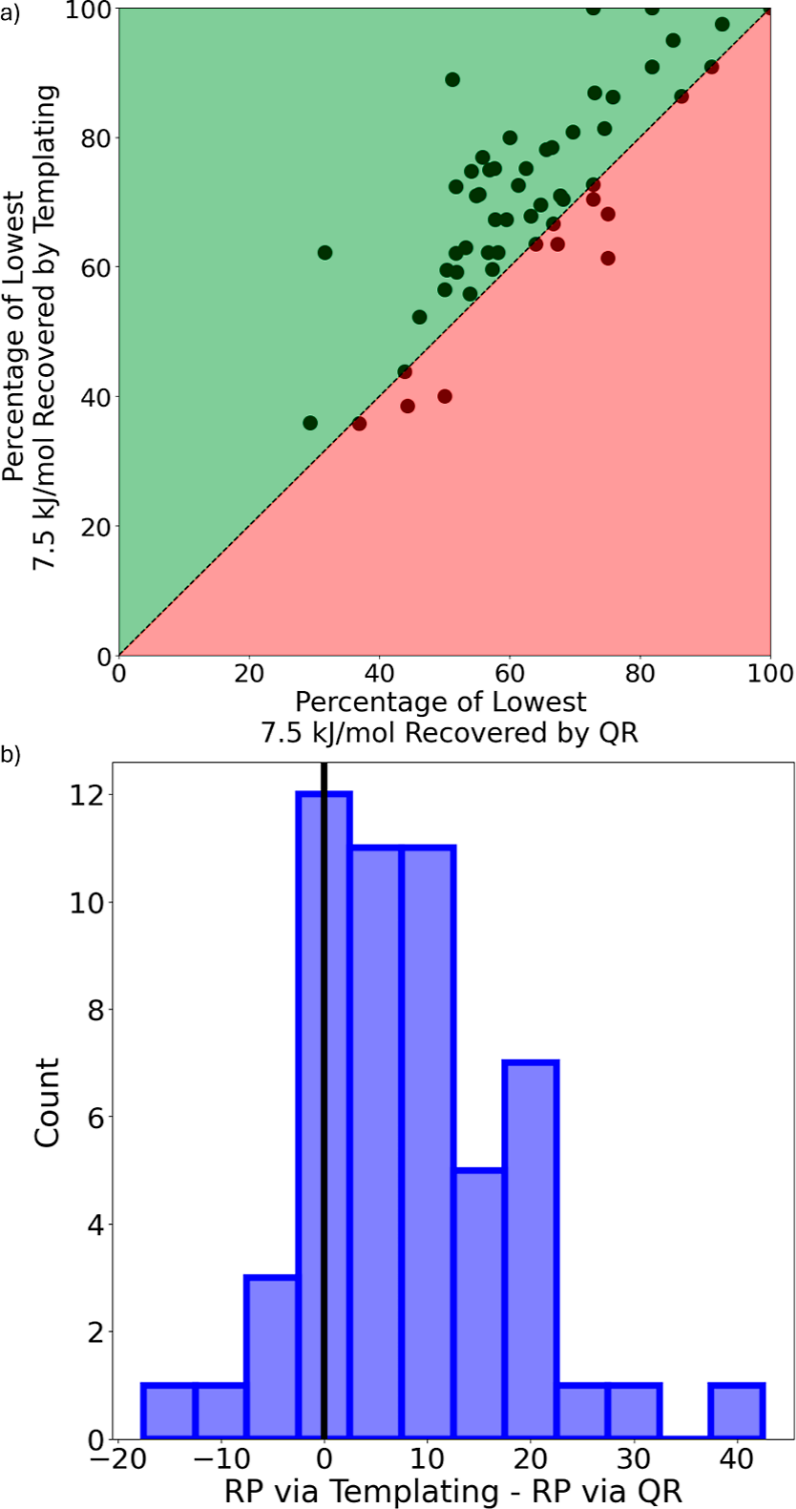
(a) Scatter plot of the RP values for each templating CSP run using 25 kJ/mol template selection window and the respective RP values for a QR CSP run using equivalent sampling. Points above the line *x* = *y* indicate superior performance of the templating approach, with greater height above the line representing greater advantage (b) histogram showing the frequency of different discrepancies between the RP values for templating CSP runs using a 25 kJ/mol template selection window and the respective equivalent QR CSP runs. Values are given by RP­(templating)—RP­(QR).

These comparisons display a clear, though not universal, trend of templating CSP outperforming QR CSP with equivalent sampling—suggesting that the guiding effect of the template landscape is impactful and thereby supporting this proof of concept. The extent of the performance gap varies. Figure 9b does show a peak centered around 0%, in which templating CSP and small QR CSP perform similarly, however the next highest peaks show improvement of templating CSP over QR CSP. Additionally, there is a total count of 37 cases demonstrating recovery of an extra 2.5–42.5% of low energy target structures using templating compared to just 5 cases of quasi random CSP recovering an extra 2.5–17.5% of low energy target structures than templating, demonstrating notable advantage of templating CSP.

To ensure that these comparisons were not unfairly biased by the strict standards used to define recovery—we ran additional checks on a small subset of the template/target pairs (one pair per family investigated). In these tests, we established “recovery” of a given structure using a looser tolerance of 0.3 Å RMSD_30_ of cluster overlay. The calculated RP values for the templating and quasi-random approaches maintained the same trend of templating CSP leading to higher recovery than small quasi-random CSP (See Section S9). We therefore conclude that the advantage of templating CSP over small quasi-random CSP is a consistent effect and not any artifact of the strict assessment.

#### Timings

We have demonstrated the advantages of templating CSP in predicting the key regions of target landscapes using small sampling. For fast CSP however, it is not only the number of sampled structures (and therefore required geometry optimizations) that is of importance, but also the computational cost of those minimizations.

We explored comparison of these minimization costs for trial structures generated via templating to those generated via quasi-random search methods. The minimization workflow used is the same minimization workflow that was used in production of the target landscapes. We use a three step minimization process as used in the GLEE^37^ workflow.

•minimization with force field + point charges
•minimization at high pressure (0.1 GPa, force field + point charges)
•minimization with force field + multipoles

The first of these minimization steps is performed using the PMIN software.^58^ The final two steps are performed via DMACRYS^50^ using a quasi-Newton–Raphson minimizer.

For the same single template–target pairs in each family as above, we measured the average CPU-time(s) for successful minimizations and compared this to the average successful minimization time from the corresponding small-QR run (Table 3).

**Table 3:** Table Indicating the Average Successful Geometry Optimization Times for Example Template/Target Pairings (One Pairing per Family Explored) and the Average Successful Geometry Optimization Times for the Respective Equivalent QR CSP runs^a^

case	avg templating min time (s)	avg QR min time (S)	time reduction (%)
NTCDA in PTCDA	11.45	13.70	16.42
BZDIOX in WARPOW	11.14	16.45	32.28
VENYUI in VENZAP	13.61	17.12	20.50
BENZEN in PRMDIN	7.02	8.63	18.66
PHTHAO in PHALIM	8.29	11.36	27.02
ETHLEN in KEMZIL	4.35	4.13	–5.08

a Each optimization was run in serial, so times provided are in both CPU seconds and real time seconds. Times are the average, across all successful optimizations, for a single trial structure to complete geometry optimization.

This showed the minimization time for trial structures generated via templating to be generally shorter than that for trial structures generated via small QR CSP, with an average decrease of 18.3% in time per structure. This is an encouraging sign for the proof of concept. Combined with the results in Figure 9, we have demonstrated that templating CSP will outperform equivalent small QR CSP—with lower minimization cost in most instances. The faster geometry optimizations suggest that templating CSP may prove advantageous over small QR for fast CSP even where the performance is similar (i.e the cases in the peak centered around 0% in 9b.) The lower minimization time may also suggest that the trial structures themselves lie closer to the minima—reinforcing the benefits of guided sampling.

We also explored the impact of altering the minimization workflow. When steps 1 and/or 2 of the minimization workflow are removed, the performance gap regarding minimization times widened (See Section S10). This reflects the more physically reasonable nature of the trial structures generated via templating, rendering the minimization of those structures less reliant upon application of external pressure to encourage close packing and initial charge-based minimization to improve the structures prior to final geometry optimization. When using a two step workflow (skipping step 2), minimization times for templating CSP decreased, meaning that the cost of the templating workflow could be decreased by using this faster minimization approach. The same cost improvements were not seen for structures generated via QR CSP. However, we reassessed the recovery percentages (RP) for the methods using these alternative minimization workflows and found that the minimization workflow impacted RP, with the nature and extent of the impact being variable between template/target pairs (See Section S10). This demonstrates that minimization workflow impacts upon the minima reached and so alteration of minimization workflows should be applied mindfully. Therefore, at this time, we do not recommend alteration to the established minimization workflow—despite the potential decrease in computational cost. It is worth noting that the target landscapes were derived using the full three-step optimization protocol, and so one cause of the changes to the RP values upon changing minimization protocol, given the strict criteria used, may simply be slight changes in the minimization workflows leading to derivation of close but different minima on the final energy surface.

At this juncture, given the nature of the work as a proof of concept, fair quantitative evaluation of the full implementation times, including the costs of structure generation and any costs associated with automation of the workflow cannot be given. Qualitative discussion of factors likely to impact implementation times and discussion of possible templating workflow improvements for reduced cost is provided in Section S11.

### Correlation of Landscapes

An additional area of interest when considering the use of previously predicted crystal structures in CSP for similar molecules is the similarity between the respective CSP landscapes. We assess this similarity by exploring the correlation between the respective CSP landscapes; that is, we assess the correlation between the energetic rankings of templates and the energetic rankings of the analogues formed from them.

This is of general interest in exploring the generalizability/commonality of factors determining the stability of molecular crystals. Notable correlation between the CSP landscapes of similar molecules would offer support for the templating approach, as it would suggest that there are similar favorable interactions for the two systems, and therefore it is more likely for similar crystal structures (i.e template/analogue crystal structure pairs) to be possible. However, while strong correlation of these energetic rankings would be of interest for the knowledge of molecular crystals and would be promising for the templating approach, it is not necessary for the method’s success. It is merely the presence of successfully predicted low energy target structures that is required for successful templating CSP. The relative ranks of the templates used to produce analogues does not impact the utility of the CSP, provided the templates are at least of suitably low energy so as to be selected for use in analogue formation.

For each template–target pair investigated, we assessed the Kendall rank correlation between the template landscapes and the corresponding deduplicated analogue landscapes. So as to explore the general correlation of landscapes, we did this for the cases using a full landscape of templates and corresponding formed analogues. Assessing this correlation requires a one-to-one mapping of structures between landscapes. For each case, we calculated the corresponding rank correlations, mapping structures between landscapes such that each template is mapped to the lowest energy analogue formed from it. All other analogues are excluded from consideration. Template structures for which no analogues were present on the final analogue landscape were excluded from the mapping and calculations. We calculated these rank correlations between the full analogue and template landscapes. The results indicated weak–moderate correlation between the CSP landscapes of similar molecules in most cases (mean: 0.255 ± 0.102, max: 0.462, Full results in Section S12). This suggests that the CSP landscapes of similar molecules are related to a degree. Though the correlation is not strong in most cases, it should be noted that strong correlation between often densely populated CSP landscapes is a tall-order and that most of the correlations noted are significant. It is natural to question whether the rank correlation between a given pair of template and analogue landscapes was related to the overall success of templating CSP for that system pairing. We made brief investigation into this possibility (see Section S14) but it did not appear to be the case, with no significant relationship between the extent of landscape correlation and the recovery of low-energy target structures for templating CSP with the full landscape or 25 kJ/mol template selection windows, and only a weak correlation for templating CSP with the 15 kJ/mol template selection window. This finding suggests that other factors dominate the success of templating in any given case.

The low correlations, despite their significance, may explain the poorer percentage recovery when performing templating CSP using templates from a 15 kJ/mol window—as some of the templates required to reach low-energy structures on the target landscape will lie higher in energy on the template landscape. However, the presence of some correlation indicates that it may be more likely for a low-energy template structure to lead to a low energy analogue. Therefore, while templating performance decreases when using a low-energy selection of templates, the efficiency may increase.

To test this, for each template/target pair, we calculated a metric we call the distribution of matches—given by the sampling efficiency of templating CSP with a restricted set of templates relative to templating CSP using all available templates. (See full results and discussion in Section S13). In almost all cases, these relative efficiency rates were greater than 1, and were notably higher when using a 15 kJ/mol selection window than when using a 25 kJ/mol selection window. This finding corroborates the suggestion that, while the correlation is insufficient for performing templating CSP using a set of low-energy templates alone, the relationship between the CSP landscapes of similar molecules means that low-energy templates have an increased likelihood of leading to a low-energy analogue. This is of note for further development of templating CSP. It may be that it is more powerful to perform templating CSP using various low-energy template crystal structures, pooled from a set of multiple template molecules, than to use a full landscape of template structures from a single template molecule. This could be investigated in further studies of templating CSP.

### Templating from Dissimilar Molecules

As a final test for the proof-of-concept study, we sought to explore the breadth of applicability of the approach. For this test, we attempted templating CSP for a template/target pair that, intuitively, appear less similar. We attempted to predict crystal structures of MEMTED using analogues of predicted NTCDA crystal structures. The MEMTED and NTCDA molecules (diagrams can be seen in Figure 1) are from different families, being neither differently sized analogues of one another, nor are they interchangeable by a small number of chemical substitutions. In this instance, there are 40 structures in the 7.5 kJ/mol window of the target landscape to recover and 5322, 996, and 152 unique analogues formed from the full landscape, 25 and 15 kJ/mol runs, respectively. The performance of templating CSP for this template/target pair (Table 4) was not the strongest case investigated. However, the known structure was predicted in all cases and the RP measured was on par with the results of some notably more similar template/target pairs. To take an example, the recovery percentage of 87.5% when using all available templates is comparable to the recovery percentages of 87.6% and 85.0% for the WARPOW/CONYAH and BZDIOX/MEMTED template/target pairs respectively (See Section S7) and the performance was above the average RP of 74.9%.

**Table 4:** Recovery of Known Structures and Recovery Percentage Performance Data for Templating CSP Case Predicting MEMTED Structures Using NTCDA Analogues

starting set	RK	RP (%)
full landscape	1 of 1	87.5
25 kJ/mol	1 of 1	47.5
15 kJ/mol	1 of 1	17.5

As an initial result, this suggests that templating between CSP landscapes of less similar molecules is worthy of investigation. One explanation for the performance in this case may be that molecular shape-based factors, such as those discussed in the box model of packing,^27^ could be dominating. Following this proof-of-concept study, further investigation will be required to corroborate and clarify the limits of this broad applicability, and to investigate approaches to identifying ideal template/target molecule pairs.

If, after further testing, the method proves successful for more dissimilar template/target molecule pairs, this will broaden the opportunities for the approach. In particular, it widens the scope for making use of the large amount of CSP data being produced and shared, as there is more likely to exist an available CSP landscape for a sufficiently suitable molecule such as to facilitate templating CSP. However, adaptations to the analogue generation approach would be needed to facilitate the overlay of molecules not sharing meaningful substructure.

One factor to note is that, while the systems differ in chemical nature, and do not have analogous shapes, there remain crucial factors of shape-similarity. For example, the NTCDA molecule is planar, and the deviation of the MEMTED molecule from planarity is minor. Both molecules could also be contained within similarly sized cuboids. The success of templating in this case could therefore be said to be in line with the box-model theory.^27^ The influence of shape-similarity on templating CSP is therefore of interest for future investigation. This may have particular relevance if applying templating CSP to flexible molecules. Templating CSP applied to flexible systems raises the question of which conformers/conformations of a target molecule to place into a template crystal. One option would be to use a randomly selected conformer or low-energy conformation of the target molecule to place into each template (or to form several analogues, each with a different target molecule conformation), in order to sample broadly and without bias. However, if shape-similarity were a crucial factor, this may be unsuccessful and an approach of forming analogues based on a target molecule conformation most similar to the template molecule in-crystal conformation could prove beneficial.

#### Summary

To aid understanding, Table 5 summarizes the main test/metrics investigated and the qualitative conclusions drawn.

**Table 5:** Metrics Explored in Order to Evaluate Templating CSP and the Proposed Qualitative Conclusions Drawn from Them

metric	finding
recovery of known structures (RK)	RK is below what would be expected from extensive QR CSP, but remains promising for fast CSP when using templates from a full landscape or 25 kJ/mol window. There were some known structures which were predictable via templating CSP with some but not all template molecules. Templating using templates from a 25 kJ/mol window predicts known structures better than QR CSP with equivalent sampling
recovery of low-energy target structures (RP)	RP is lower than for extensive QR CSP, but remains promising for fast CSP when using templates from a full landscape or 25 kJ/mol window. Templating using templates from a 25 kJ/mol window usually outperforms QR CSP with equivalent sampling
relation of RP to prediction task difficulty	RP values for a templating task are moderately and significantly correlated with the difficulty of the task based on the ratio of targets to sample structures. This will be a key factor in the success of a prediction task, but is insufficient to explain performance in isolation
comparison of minimisation times relative to small QR CSP	successful lattice energy minimizations are faster for trial structures generated via templating than via QR
correlation between energetic rankings of template and analogue landscapes	the energetic rankings of templates and the analogues formed from them are weakly but significantly correlated. Performing templating CSP from low-energy crystal structures of one template molecule is insufficient but the sampling efficiency may be higher

## Conclusions

We have developed and evaluated a proof-of-concept for a fast, approximate CSP method in which trial structures are generated by analogy to previously predicted crystal structures of similar molecules. We assessed the potential of the method with regard to its ability to predict known experimental structures, and to predict the crucial low energy region of CSP landscapes that would have been predicted via traditional CSP approaches (using a quasi-random search method). We compared our results to those that would be expected if using conventional QR CSP methods with reduced sampling, including comparison of the cost of geometry optimization of trial structures. We also investigated the correlation between the CSP landscapes of similar molecules and explored the limits of templating CSP via investigation of a more dissimilar template/target molecule pair.

The initial study suggests promise of the templating CSP method, warranting further investigation. We found that, given a suitable selection of initial templates (a full landscape or 25 kJ/mol window selection for templates for some given template molecule), templating CSP was able to predict the known crystal structures for 84% of targets. This is an improvement over the use of QR CSP with minimal sampling, which achieved full recovery for 68% of targets.

Recovery of the low energy region of a target CSP landscape was also able to reach respectable figures, with average RP values of 74.9% when using a full landscape of template structures, or 70.6% when using templates lying in a 25 kJ/mol window. This performance when using a 25 kJ/mol window of template structures demonstrated the potential of templating CSP, which outperformed QR CSP with equivalent sampling—recovering at least an extra 2.5% of the low-energy target structures in 69% of trialled cases, with an average (across these cases) extra 12.8% of the low-energy target landscapes recovered.

These figures suggest that templating CSP holds promise as a method of fast, if less thorough CSP. The approach would not be appropriate for all purposes, for example not being suited to CSP being used to assess the risk of a more stable structure of a pharmaceutical, as in such a CSP it would be crucial to recover all crystal structures corresponding to low-lying local energy minima on the energy landscape. However, methods that call for a fast assessment of crystal packing possibilities are emerging, opening up new approaches for materials discovery, where speed, rather than completeness, of CSP is the crucial factor. Our findings that templating CSP can outperform traditional CSP with equivalently minimal sampling suggest that the approach could be implemented in these applications to increase the performance of the structure prediction while maintaining speed.

Additionally, the average times for successful geometry optimizations were shorter for trial structures generated via templating CSP than quasi-random CSP, offering further advantage of the approach for fast, approximate CSP and implying a greater initial proximity to minima that adds credence to the templating method.

Performance of templating CSP did vary with the energy window used to extract templates, with results indicating that selecting templates from a 15 kJ/mol window on the original landscapes is unlikely to be sufficient for effective templating CSP. A key factor in this is likely the extent of the sampling. However, we demonstrated that the extent of sampling was not the sole determiner of success.

Comparison of template and analogue landscapes found low, but statistically significant, correlation between the energetic rankings of templates and the analogues formed from them. This finding confirms the existence of a relationship between CSP landscapes of similar molecules, but also highlights the limits of this relationship—which are reflected in the poorer performance of templating CSP when using a 15 kJ/mol template selection window. However, the presence of a significant correlation, coupled with our finding of increased sampling efficiency when using a low-energy template selection window suggests a possibility of improving templating CSP by pooling low-energy template crystal structures from different systems.

Lastly, testing the limits of the approach—applying it to a more dissimilar template/target pair and demonstrated that a reasonable degree of success could still be achieved, for example recovering 87.5% of low-energy target structures when using all available templates, which is on a par with, among other examples, the recovery percentage of 87.6% the WARPOW/CONYAH template/target pair. This suggests that the approach may be more widely applicable than initially expected, if suitable modifications to the method are made. Further investigations should explore the importance of shape-similarity factors in determining suitability of possible template/target molecule pairs.

Overall, we believe that this proof of concept study indicates that templating CSP is a promising approach for fast crystal structure prediction. We acknowledge, however, that the initial testing set of six families of small, rigid molecules is limited. In future testing, data on a larger number of systems will be required in order to corroborate results and to meaningfully investigate the factors influencing the success of templating for a given template/target molecule pair. Additionally, as the CSP field progresses, methods are tasked with predicting crystal structures of larger, flexible molecules.^7^ The promise of templating CSP for such systems should be assessed. Further investigation should also explore optimization of the method followed by deeper quantification of the computational costs.

## Supplementary Material



## Data Availability

Codes for templating CSP is available at: https://gitlab.com/mol-cspy/mol-cspy Crystal structures predicted via extensive quasi random sampling (NTCDA,PTCDA, and XUGHUD2), crystal structures predicted via templating CSP for each template/target pair using all available crystal structures of the template molecule, crystal structures predicted via restrictive sampling quasi-random CSP, all low energy target landscapes, and SMILES strings of similar molecule families will be available at: 10.5258/SOTON/D3900

## Notes

Additional data plots and results; Details of Crystal structure prediction and known structure match identification; Extended methodological details; Qualitative discussion of factors influencing implementation times (PDF)
