# Supplementary material for: Fast Molecular Crystal Structure Prediction Using Sampling by Analogy to Previously Predicted Landscapes
Source: J Chem Theory Comput. 2026 Jun 9;22(12):6239–53. doi: 10.1021/acs.jctc.6c00596 (PMC13296494; doi:10.1021/acs.jctc.6c00596)
Supplement: Supplementary file 1 [file ct6c00596_si_001.pdf]

Fast Molecular Crystal Structure Prediction using Sampling by Analogy to Previously Predicted Landscapes

Supporting Information

Jennie Martin[1] and Graeme M. Day[1]

[1]School of Chemistry and Chemical Engineering, University of Southampton, Southampton, SO17 1BJ, United Kingdom

Contents

1 Crystal Structure Prediction of NTCDA, PTCDA and XUGHUD2 **2**

2 Completeness of Target Landscapes **4**

3 Identification of Similar Molecule Families **7**

4 Identification of Overlays **9**

5 Experimental Structures used for Assessing Recovery of Known Structures **13**

6 Recovery of Known Structures **15**

7 Recovery of Low Energy Target Structures **20**

8 Additional Graphs of Recovery Percentage per Family **23**

9 Recovery of Low Energy Target Structures with Looser Criterion **25**

10 Minimisation Timings and Performance with Alternative Workflows **27**

11 Factors Influencing Implementation Time **32**

12 Landscape Correlations **33**

13 Distribution of Matches **36**

14 Landscape Correlation vs Performance **39**

1 Crystal Structure Prediction of NTCDA, PTCDA and XUGHUD2

To obtain a target landscape for XUGHUD2, prediction crystal structured prediction was performed in-house, following the same workflow used to obtain the most of the remaining target landscapes (except for NTCDA and PTCDA). Details are not given here, but can be seen in Ref [1].
To obtain target landscapes for NTCDA and PTCDA, crystal structured predictions were performed in-house, following a workflow closely aligned to that used to obtain the remaining target landscapes [1].

For each system, a quasi-random CSP search was performed using the Global Lattice Energy Explored (GLEE) [2] protocol discussed in the main text, to predict Z'=1 crystal structures of the molecule in a single conformation.

The conformation used in each case was that obtained from a DFT-level (B3LYP [3–5] + GD3BJ [6]/6-311G**[7]) gas-phase geometry optimisation performed using Gaussian09 [8]. Quasi-randomly generated trial structures were lattice-energy minimised using the three step process outlined in the main text, and using interatomic forcefield FIT [9] with distributed atomic multipoles calculated at DFT (PBE0[10, 11]/6-31G** [12–14]) level.

For each system, structure generation and optimisation continued until 10,000 fully optimised crystal structures had been obtained in each of the ten most common space groups in the CSD[15] for organic molecules ($P12_1/c1, P2_12_12_1, P\bar{1}, P12_11, Pbca, C12/c1, Pna2_1, C121$).

The resulting landscapes were de-duplicated using comparison of pXRD patterns followed by use of the COMPACK [16] algorithm via the CSD API [17].

To evlaute the success of the prediction, the landscapes were searched for predicted structures corresponding to known experimental crystal structures of the molecules. Matches were assessed via geometric overlays in the CSD API [17], and a predicted structure was said to match an experimental structure if 30/30 molecules of 30 molecule clusters of the repsective crystal structures could be overlaid within tolerances of 20% for distances and 0.2° for angles. The experimental structures used are those indicated by the following CSD refcodes: NTCDA - KENDEM [18], PTCDA (α) - SUWMIG02 [19] , PTCDA (β) - SUWMIG03 [19], XUGHUD2 - XUGHUD01 (edited to remove additional hydrogen). The predicted structure/experimental structure matches

for each system and polymorph are indicated in S1.

System	No. Unique Structures	Energetic Ranking of Match	$RMSD_{30}$ of Match (Å)
NTCDA	672	1	0.336
PTCDA	1186	1 (α), 2 (β)	0.376 (α), 0.360 (β)
XUGHUD2	5042	118	0.331

Table. S1: Results of crystal structure prediction for NTCDA,PTCDA and XUGHUD2

2 Completeness of Target Landscapes

Table S2 shows the number of low energy structures ($\leq$ 7.5 kJ/mol) from the corresponding templating CSP, using a full landscape of templates, which were not present at any energy on the original target landscape. To ensure the tests were not biased by the templating landscapes being less thoroughly de-duplicated than the target landscapes, the templating CSP landscapes were deduplicated using a deduplication algorithm inspired by the COMPACK method[16], as was implemented for derivation of most target landscapes[1]. A structure was assessed to be present on the target landscape if 30/30 molecules of representative clusters of the target structure and the structure recovering it could be overlaid to within tolerances of 20% for distances and 20° for angles, regardless of the resulting $RMSD_{30}$.

Case	New Structures
BENZEN in PRMDIN	0
BENZEN in CILWUP	0
CILWUP in BENZEN	0
CILWUP in PRMDIN	0
PRMDIN in BENZEN	0
PRMDIN in CILWUP	0
PHATHAO in PHALIM	0
PHATHAO in INDDON	0
PHATHAO in TIPVIZ	0
PHATHAO in XUGHUD1	0
PHALIM in PHATHAO	0
PHALIM in INDDON	0
PHALIM in TIPVIZ	0
PHALIM in XUGHUD1	1
INDDON in PHATHAO	4
INDDON in PHALIM	4
INDDON in TIPVIZ	2
INDDON in XUGHUD1	4
TIPVIZ in PHATHAO	0
TIPVIZ in PHALIM	0
TIPVIZ in INDDON	0
TIPVIZ in XUGHUD1	0

XUGHUD1 in PHATHAO	0
XUGHUD1 in PHALIM	0
XUGHUD1 in INDDON	0
XUGHUD1 in TIPVIZ	0
ETHLEN in KEMZIL	6
KEMZIL in ETHLEN	3
PHATHAO in XUGHUD2	0
PHALIM in XUGHUD2	0
INDDON in XUGHUD2	5
TIPVIZ in XUGHUD2	1
XUGHUD2 in PHATHAO	0
XUGHUD2 in PHALIM	0
XUGHUD2 in INDDON	0
XUGHUD2 in TIPVIZ	0
XUGHUD1 in XUGHUD2	0
XUGHUD2 in XUGHUD1	0
BZDIOX in CONYAH	5
BZDIOX in WARPOW	2
CONYAH in MEMTED	1
CONYAH in BZDIOX	0
CONYAH in WARPOW	2
MEMTED in CONYAH	2
MEMTED in WARPOW	0
WARPOW in BZDIOX	0
WARPOW in CONYAH	2
WARPOW in MEMTED	0
VENYUI in VENZAP	0
VENZAP in VENYUI	0
MEMTED in BZDIOX	3
BZDIOX in MEMTED	5
NTCDA in PTCDA	0
PTCDA in NTCDA	0

Table. S2: Number of low-energy structures ($\leq$ 7.5 kJ/mol) from templating CSP, using a full landscape of templates, that are not present on the respective target landscapes

3 Identification of Similar Molecule Families

We identified families of similar molecules for potential investigation of templating CSP, by filtering of a set of SMILES strings corresponding to a large dataset containing the CSP landscapes over over 1000 small organic molecules [1].

We developed a search of the SMILES strings that identified whether a given pair of molecules could be interchanged by 3 or fewer single heavy-atom chemical substitutions along the backbone of the molecule. The addition/removal of further hydrogen atoms, such as the substitution of a nitrogen atom for a CH group would also be permitted by the search. This was assessed by comparison of the strings to identify altered characters. First, SMILES string pairs were filtered to ensure all considered pairs were of the same length. The search was then based on position-by-position comparison of the strings, with the number of changes to alphabetical characters being noted. If the number of changes was 3 or fewer, the pair were considered as a possibility of being an interchangeable pair. Lastly, the strings were compared after the removal of the altered alphabetical characters and considered interchangeable only if the resulting strings were identical - this prevents the possibility of unwanted changes such as addition/removal of double-bonds. The restrictions upon string length prevented the identification of molecule pairs interchangeable by the replacement of ‘unwritten’ hydrogen atoms with heavy atoms. It further prohibits replacement of single atoms with any groups represented by multiple characters.

It should be noted that the search described here is non-exhaustive, for example failing to identify molecular pairs interchangeable by exchange of a hydrogen with a heavy atom. The non-canonical nature of the SMILES strings used may also lead to strings being identified as dissimilar due to changes in the representation. The position-by-position comparison and length constraints theoretically pose an issue for multi-character elements such as chlorine. However, the literature set used contains systems composed only of C,H,N,O,and F, and so the limitation was not of concern for this work. The search was therefore considered appropriate for this work, which do not need exhaustive identification of all similar molecules.

From the SMILES comparison search, a pairwise matrix was constructed classifying each pair of molecules as either interchangeable or non-interchangeable. A Reverse Cuthill-McKee algorithm [20] implemented in SciPy [21], which attempted to rearrange the pairwise matrix so as to best group interchangeable families of molecules along the diagonal of the matrix. This led to the identification of 95 molecule families. The set of possible families was then manually searched to

select a diverse range of molecule families to study.

4 Identification of Overlays

To identify all valid overlays for a given template/target molecule pair, we first identify the maximum common substructure, using functionality within the CSD API [17]. This identifies the largest substructure that is found in both molecules. This substructure must be a single connected (i.e bonded) structure, but the bond-types are not considered. That is, it is equivalent to identifying the largest shared connected subgraph of the two molecule's graphs - if in one molecule a given graph edge corresponds to a double-bond and in the other it corresponds to a single-bond, the substructure is still counted. This 'bond-blindness' is enforced to make the process impartial to small differences in the molecular representation, for example in the representation of aromatic rings.

Then, all valid ways to overlay the template/target molecules so as to overlay the maximum common substructure must be identified. In this work , we call these 'isomorphic overlays'. There are two factors to consider:

- Multiple instances of the substructure within one/both of the molecules
- Symmetry of the substructure itself leading to multiple 'mappings' by which to equivalently overlay the substructure

Multiple instances of the same substructure within one molecule are common, and can lead to the construction of different analogues. For example, the shared C_5H_5 substructure of benzene and pyridine, appears 6 times in the benzene molecule and replacing benzene molecules in a crystal with pyridine according to these different possible overlays will result in different analogues, in which the pyridine molecule is (non-equivalently) rotated.

Symmetry of the underlying substructure also leads to multiple possible overlays, and if one or both of the whole molecules is not symmetric under the same transformation as the substructure, the different overlays can lead to the construction of different analogues. For example, in the case of the BZDIOX/MEMTED template target pair, the maximum substructure has rotational symmetry, and so when determining 'mappings' for the overlay, i.e which atom in the target molecule substructure instance to overlay with each atom in the template molecule substructure instance, there are two equally valid mappings. One can imagine these mappings as being the overlay of the substructure instances before and after rotation (or reflecting one of the instances along its mirror plane). However, whilst the substructure overlays are directly equivalent, in practice the second overlay can only be achieved via rotation one of the molecules, which results in different analogue crystal structures due to the non-planarity of the MEMTED molecule. The MEMTED molecule,

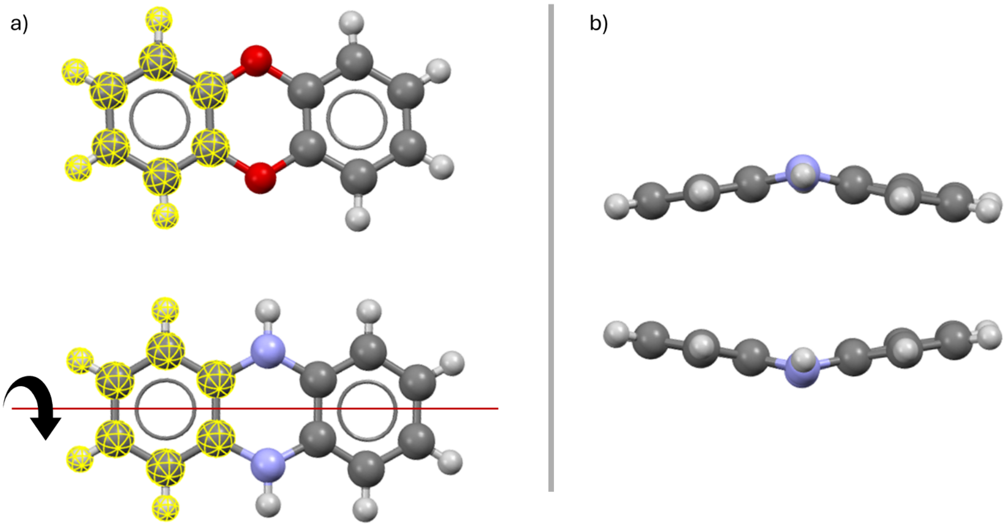

Fig. S1: a) BZDIOX and MEMTED molecules with the shared susbtructure highlighted in yellow, and the rotational symmetry of the substructure indicated in the lower (MEMTED) example. b) Side-view of the MEMTED molecule before and after rotation along the axis indicated in a)

whilst strictly sharing the mirror plane exhibited by the substructure, does not share the rotational symmetry. (See Figure S1)

There are, however, limitations to this approach to identifying valid overlays. First, it requires that a given pair of molecules share substructure. And that that substructure represent a large enough fragment of the molecules so as to reasonably guide overlay (i.e a single atom or pair of atoms, is unlikely to be important substructure for most molecules). Additionally, the identification of common substructure, substructure instances, and valid overlays, is based upon molecular graphs - i.e only connectivity is considered, and the impact of 3D geometry is ignored.

This can cause issues if the substructure instances for a given identified 'valid overlay' cannot be reasonably overlaid in 3D space. That is, that the process does identify all valid overlays, but can risk also identifying some invalid overlays. For example, in Figure S2, the identified substructure overlay (i.e if orange-indexed atom 1 in the first molecule was to overlay with orange-indexed atom 1 in the second molecule etc.) corresponds to an overlaying of substructure instances that - according to their connectivity - match. However, attempting to perform the overlay will not align the molecules in the intuitive way (i.e it will not align the molecules according to their 'similar parts') [22]

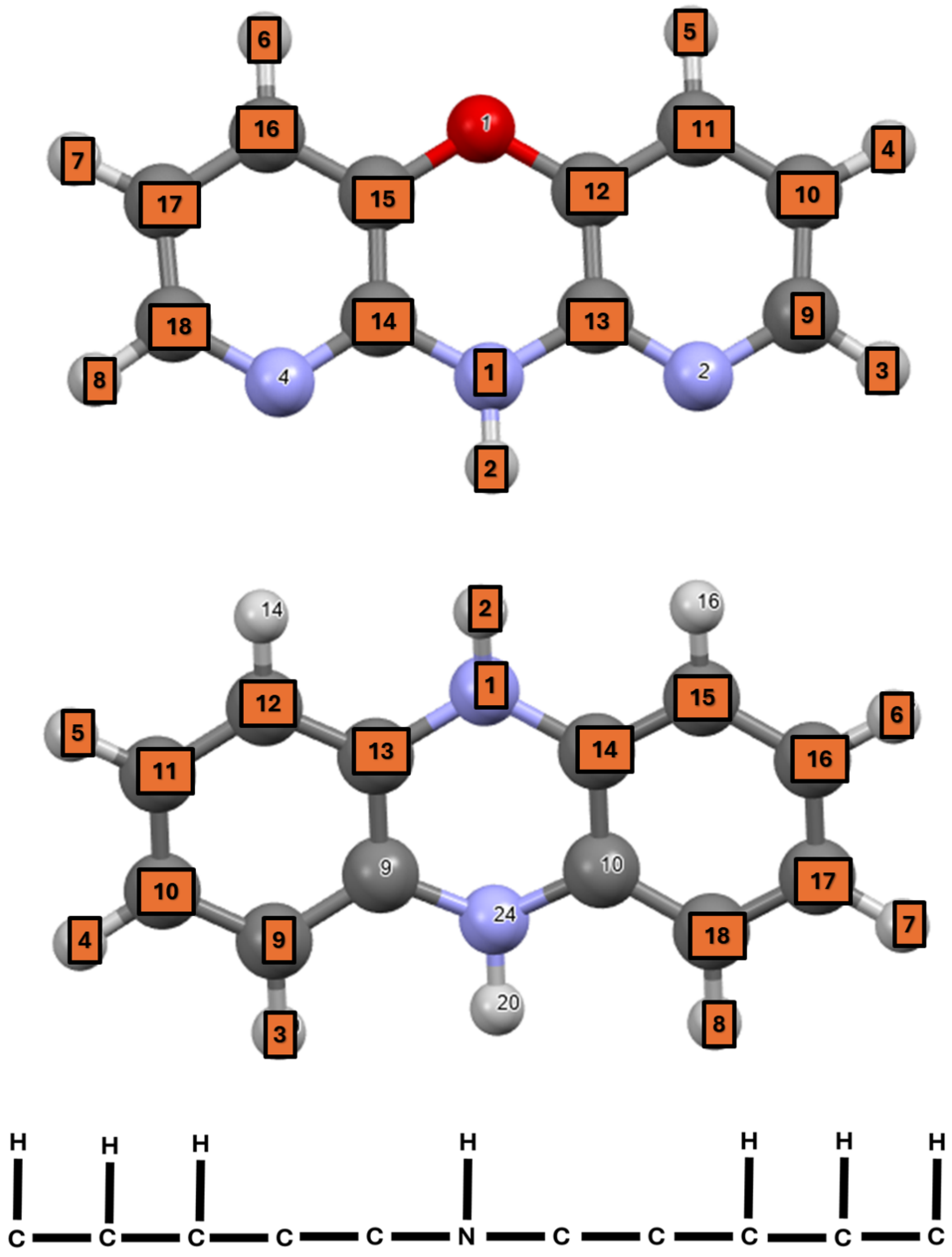

Fig. S2: Example of an identified ‘valid overlay’ - as referenced by overlaying the atoms of matching index - that does not result in a reasonable analogue. Reproduced with permission from ref [22], Copyright 2025, Jennie Martin.

Further, the identification of ‘isomorphic overlays’ based upon the symmetry of the substructure can also cause issues. Figure S3 offers insight into this problem. Due to mirror-plane symmetry of the substructure, multiple ‘valid overlays’ are identified to overlay the instances, this results in attempting to overlay (part of) the substructure according to the orange indices shown in the image. However, in order to best achieve overlay of these atoms- one molecule must be flipped. For some molecules, this would not be problematic, however, as this case (VENYUI/VENZAP) is of molecules ‘capped’ at both ends, flipping a molecule to reasonably overlay the atoms indicated , means the rest of the substructure cannot be simultaneously overlaid to within reasonable tolerances.[22]

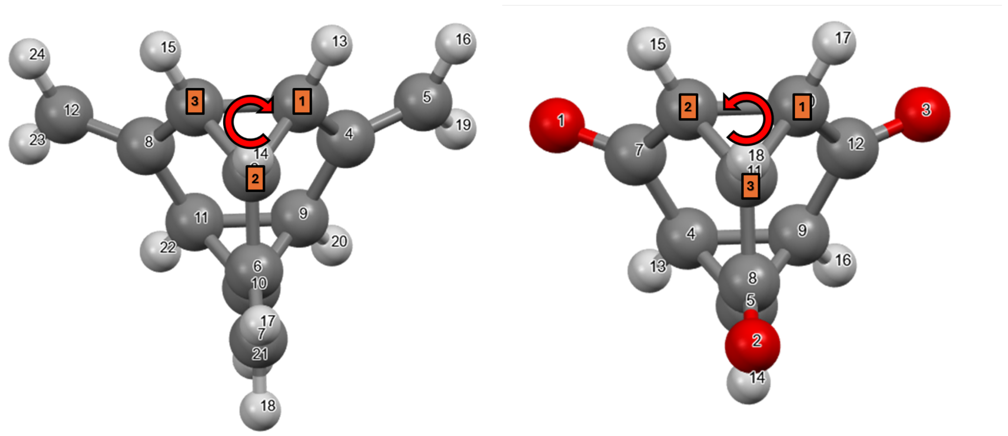

Fig. S3: Figure highlighting part of an identified ‘valid overlay’ - as referenced by overlaying the atoms of matching orange index - that is not reasonably achievable in 3D space . Reproduced with permission from ref [22], Copyright 2025, Jennie Martin.

In this work, analogues that would have been constructed for invalid overlays are rejected at the molecular overlay stage - as the analogue construction was limited to only proceed if the RMSD of the molecular overlay was less than or equal to 2 Å .The RMSD cut-off criterion applied was selected to alleviate the issue for the cases relevant to the molecules explored but, in further development, alternative approaches or a more rigorous determination of a suitable criterion may be needed.

5 Experimental Structures used for Assessing Recovery of Known Structures

For each target molecule explored, we assessed the recovery of its known crystal structures. For each target molecule, we tested the recovery of a crystal structure from the CSD [15] representing each known polymorph, provided that there was such a structure that satisfied the following requirements:

- Z' ≤ 1
- Contains only the target molecule (no ions, solvent, etc.)
- No disorder
- All 3D co-ordinates defined
- R-Factor $\leq 10\%$
- No errors
- Not polymeric

Table S3 shows the CSD refcodes of the known structures used in assessment of CSP for each target molecule.

Target Molecule	Known Structures
NTCDA	KENDEM
PTCDA	SUWMIG02, SUWMIG03
BZDIOX	BZDIOX02
CONYAH	CONYAH
MEMTED	MEMTED
WARPOW	WARPOW
VENYUI	VENYUI
VENZAP	VENZAP
BENZEN	BENZEN, BENZEN03
PRMDIN	PRMDIN20, PRMDIN
CILWUP	CILWUP11
INDDON	INDDON
PHALIM	PHALIM04

PHATHAO	PHATHAO, PHATHAO02
TIPVIZ	TIPVIZ
XUGHUD1	XUGHUD, FALHEI02
XUGHUD2	XUGHUD01 (edited to remove additional hydrogen)
ETHLEN	ETHLEN10
KEMZIL	KEMZIL01

Table. S3: CSD[15] REFCODES of known structures used to assess CSP for each target molecule

6 Recovery of Known Structures

Table S4 shows the recovery of known structures (RK) for templating CSP for each template/target molecule pairing and for different template selection windows. The performance from 'Small QR' (quasi-random CSP performed requesting the same number of crystal structures in each space-group as there were unique initial analogues in that spacegroup for templating CSP using a 25 kJ/mol template selection window and the corresponding template/target molecule pair) is also shown. A known structure is defined as recovered if there exists a predicted structure for which 30/30 molecules of 30 molecule clusters of the predicted and known crystal structures can be over-laid to within tolerances of 30% for distances and 30° for angles, regardless of the $RMSD_{30}$ of the overlay.

Table S5 shows the 'quality' of the matches between structures predicted via templating CSP (or small QR) and the known crystal structures. The $RMSD_{30}$ values shown in each case are those corresponding the lowest $RMSD_{30}$ match between the known polymorph and a predicted structure from the corresponding CSP run.

Case	RK<br>Full Landscape	RK<br>25 kJ/mol	RK<br>Small QR	RK<br>15 kJ/mol
NTCDA in PTCDA	1 of 1	1 of 1	1 of 1	1 of 1
PTCDA in NTCDA	2 of 2	2 of 2	2 of 2	2 of 2
BZDIOX in CONYAH	1 of 1	1 of 1	1 of 1	1 of 1
BZDIOX in WARPOW	1 of 1	1 of 1	1 of 1	1 of 1
CONYAH in MEMTED	1 of 1	1 of 1	1 of 1	1 of 1
CONYAH in BZDIOX	1 of 1	1 of 1	1 of 1	1 of 1
CONYAH in WARPOW	1 of 1	1 of 1	1 of 1	0 of 1
MEMTED in CONYAH	1 of 1	1 of 1	1 of 1	1 of 1
MEMTED in WARPOW	1 of 1	1 of 1	1 of 1	0 of 1
WARPOW in BZDIOX	0 of 1	0 of 1	1 of 1	0 of 1
WARPOW in CONYAH	1 of 1	1 of 1	1 of 1	1 of 1
WARPOW in MEMTED	1 of 1	1 of 1	1 of 1	0 of 1
MEMTED in BZDIOX	1 of 1	1 of 1	1 of 1	1 of 1
BZDIOX in MEMTED	1 of 1	1 of 1	1 of 1	0 of 1
VENYUI in VENZAP	1 of 1	1 of 1	1 of 1	1 of 1
VENZAP in VENYUI	1 of 1	1 of 1	1 of 1	1 of 1

BENZEN in PRMDIN	2 of 2	2 of 2	2 of 2	2 of 2
BENZEN in CILWUP	2 of 2	2 of 2	2 of 2	2 of 2
CILWUP in BENZEN	1 of 1	1 of 1	1 of 1	1 of 1
CILWUP in PRMDIN	1 of 1	1 of 1	1 of 1	1 of 1
PRMDIN in BENZEN	1 of 2	1 of 2	1 of 2	1 of 2
PRMDIN in CILWUP	1 of 2	1 of 2	1 of 2	1 of 2
PHTHAO in PHALIM	1 of 2	1 of 2	1 of 2	1 of 2
PHTHAO in INDDON	1 of 2	1 of 2	2 of 2	1 of 2
PHTHAO in TIPVIZ	2 of 2	2 of 2	1 of 2	1 of 2
PHTHAO in XUGHUD1	2 of 2	2 of 2	1 of 2	2 of 2
PHALIM in PHTHAO	1 of 1	1 of 1	0 of 1	1 of 1
PHALIM in INDDON	1 of 1	1 of 1	0 of 1	1 of 1
PHALIM in TIPVIZ	1 of 1	1 of 1	0 of 1	1 of 1
PHALIM in XUGHUD1	1 of 1	1 of 1	0 of 1	1 of 1
INDDON in PHTHAO	1 of 1	1 of 1	1 of 1	1 of 1
INDDON in PHALIM	1 of 1	1 of 1	1 of 1	1 of 1
INDDON in TIPVIZ	1 of 1	1 of 1	0 of 1	0 of 1
INDDON in XUGHUD1	0 of 1	0 of 1	0 of 1	0 of 1
TIPVIZ in PHTHAO	1 of 1	1 of 1	0 of 1	1 of 1
TIPVIZ in PHALIM	1 of 1	1 of 1	0 of 1	1 of 1
TIPVIZ in INDDON	0 of 1	0 of 1	0 of 1	0 of 1
TIPVIZ in XUGHUD1	1 of 1	1 of 1	0 of 1	1 of 1
XUGHUD1 in PHTHAO	1 of 2	1 of 2	1 of 2	1 of 2
XUGHUD1 in PHALIM	1 of 2	1 of 2	0 of 2	0 of 2
XUGHUD1 in INDDON	1 of 2	1 of 2	1 of 2	1 of 2
XUGHUD1 in TIPVIZ	1 of 2	1 of 2	1 of 2	1 of 2
PHTHAO in XUGHUD2	2 of 2	2 of 2	2 of 2	2 of 2
PHALIM in XUGHUD2	1 of 1	1 of 1	0 of 1	1 of 1
INDDON in XUGHUD2	1 of 1	1 of 1	1 of 1	0 of 1
TIPVIZ in XUGHUD2	1 of 1	1 of 1	0 of 1	1 of 1
XUGHUD2 in PHTHAO	1 of 1	1 of 1	0 of 1	1 of 1
XUGHUD2 in PHALIM	1 of 1	1 of 1	0 of 1	1 of 1
XUGHUD2 in INDDON	0 of 1	0 of 1	0 of 1	0 of 1

XUGHUD2 in TIPVIZ	1 of 1	1 of 1	0 of 1	1 of 1
XUGHUD1 in XUGHUD2	1 of 2	0 of 2	0 of 2	0 of 2
XUGHUD2 in XUGHUD1	0 of 1	0 of 1	0 of 1	0 of 1
ETHLEN in KEMZIL	1 of 1	1 of 1	1 of 1	1 of 1
KEMZIL in ETHLEN	0 of 1	0 of 1	0 of 1	0 of 1

Table. S4: Recovery of known structures (RK) for templating CSP for each template/target molecule pairing for different template selection windows and for quasi-random CSP with sampling equivalent to that used in templating CSP with the corresponding template/target pair and a 25 kJ/mol template selection window

Case	Best match $RMSD_{30}$ Full Landscape (Å)	Best match $RMSD_{30}$ 25 kJ/mol (Å)	Best match $RMSD_{30}$ Small QR(Å)	Best match $RMSD_{30}$ 15 kJ/mol (Å)
NTCDA in PTCDA	0.335	0.336	0.337	0.336
PTCDA in NTCDA	0.375, 0.362	0.375, 1.439	0.376, 0.355	1.504, 1.439
BZDIOX in CONYAH	0.093	0.093	0.104	0.093
BZDIOX in WARPOW	0.093	0.093	0.104	0.093
CONYAH in MEMTED	0.326	0.326	0.326	0.326
CONYAH in BZDIOX	0.525	0.525	0.326	0.525
CONYAH in WARPOW	0.326	0.326	0.525	None
MEMTED in CONYAH	0.38	0.38	0.381	0.381
MEMTED in WARPOW	0.381	0.381	0.38	None
WARPOW in BZDIOX	None	None	0.565	None
WARPOW in CONYAH	0.565	0.565	0.565	0.565
WARPOW in MEMTED	0.567	0.567	0.565	None
MEMTED in BZDIOX	0.38	0.38	0.381	0.38
BZDIOX in MEMTED	0.104	0.104	0.104	None
BENZEN in PRMDIN	0.293, 0.415	0.293, 0.415	0.293, 0.416	0.293, 0.415
VENYUI in VENZAP	0.099	0.099	0.099	0.099
VENZAP in VENYUI	0.218	0.866	0.217	0.219
BENZEN in CILWUP	0.293, 0.415	0.293, 0.415	0.293, 0.416	0.293, 0.415
CILWUP in BENZEN	0.351	0.351	0.351	0.351

CILWUP in PRMDIN	0.351	0.351	0.351	0.351
PRMDIN in BENZEN	0.299, None	0.299, None	0.299, None	0.299, None
PRMDIN in CILWUP	0.732, None	0.732, None	0.299, None	0.732, None
PHTHAO in PHALIM	None, 0.963	None, 0.963	None, 0.947	None, 0.963
PHTHAO in INDDON	0.165, None	0.165, None	0.168, 0.947	0.165, None
PHTHAO in TIPVIZ	0.165, 0.946	0.165, 0.946	None, 0.947	None, 0.946
PHTHAO in XUGHUD1	0.167, 0.946	0.167, 0.946	None, 0.947	0.167, 0.947
PHALIM in PHTHAO	0.205	0.205	None	0.205
PHALIM in INDDON	0.205	0.205	None	0.205
PHALIM in TIPVIZ	0.205	0.205	None	0.205
PHALIM in XUGHUD1	0.206	0.206	None	0.206
INDDON in PHTHAO	0.095	0.096	0.096	0.096
INDDON in PHALIM	0.096	0.096	0.096	0.096
INDDON in TIPVIZ	0.096	0.096	None	None
INDDON in XUGHUD1	None	None	None	None
TIPVIZ in PHTHAO	0.644	0.644	None	0.644
TIPVIZ in PHALIM	0.644	0.644	None	0.644
TIPVIZ in INDDON	None	None	None	None
TIPVIZ in XUGHUD1	0.644	0.644	None	0.644
XUGHUD1 in PHTHAO	None, 0.793	None, 0.792	None, 0.792	None, 0.792
XUGHUD1 in PHALIM	None, 0.793	None, 0.793	None, None	None, None
XUGHUD1 in INDDON	None, 0.793	None, 0.793	None, 0.792	None, 0.793
XUGHUD1 in TIPVIZ	None, 0.794	None, 0.794	None, 0.792	None, 0.794
PHTHAO in XUGHUD2	0.166, 0.946	0.167, 0.946	0.168, 0.947	0.164, 0.946
PHALIM in XUGHUD2	0.206	0.206	None	0.206
INDDON in XUGHUD2	0.096	0.096	0.096	None
TIPVIZ in XUGHUD2	0.644	0.644	None	0.644
XUGHUD2 in PHTHAO	1.151	1.151	None	1.151
XUGHUD2 in PHALIM	0.331	0.331	None	0.331
XUGHUD2 in INDDON	None	None	None	None
XUGHUD2 in TIPVIZ	0.331	0.331	None	0.331
XUGHUD1 in XUGHUD2	None, 0.793	None, None	None, None	None, None
XUGHUD2 in XUGHUD1	None	None	None	None

ETHLEN in KEMZIL	0.21	0.21	0.209	0.21
KEMZIL in ETHLEN	None	None	None	None

Table. S5: ‘Quality’ of the matches between predicted structures and the known crystal structures for templating CSP for each template/target molecule pairing with different template selection windows and for quasi-random CSP with sampling equivalent to that used in templating CSP with the corresponding template/target pair and a 25 kJ/mol template selection window.The $RMSD_{30}$ values shown in each case are those corresponding the lowest $RMSD_{30}$ match between the known polymorph and a predicted structure from the corresponding CSP run.

7 Recovery of Low Energy Target Structures

Table S6 shows the number of low-energy target structures for each target molecule.

Target Molecule	No. Structures
NTCDA	2
PTCDA	10
BZDIOX	307
CONYAH	153
MEMTED	40
WARPOW	137
VENYUI	9
VENZAP	22
BENZEN	102
PRMDIN	178
BENZEN	241
INDDON	87
PHALIM	125
PHATHAO	62
TIPVIZ	11
XUGHUD1	44
XUGHUD2	52
ETHLEN	457
KEMZIL	325

Table. S6: Number of low-energy ($\leq$ 7.5 kJ/mol) structures on each target landscape

Table S7 shows the percentage of low energy ($\leq$ 7.5 kJ/mol) target structures recovered (RP) for templating CSP for each template/target molecule pairing and for different template selection windows. The performance from 'Small QR' (quasi-random CSP performed requesting the same number of crystal structures in each spacegroup as there were unique initial analogues in that spacegroup for templating CSP using a 25 kJ/mol template selection window and the corresponding template/target molecule pair) is also shown. A target structure is defined as recovered if there exists a predicted structure for which 30/30 molecules of 30 molecule clusters of the predicted and known crystal structures can be overlaid to within tolerances of 20% for distances and 20° for angles such that the $RMSD_{30}$ of the overlay is less than or equal to 0.05Å.

Case	RP Full Landscape	RP 25 kJ/mol	RP 15 kJ/mol	RP Small QR
NTCDA in PTCDA	100.0	100.0	100.0	100.0
PTCDA in NTCDA	70.0	40.0	20.0	50.0
BZDIOX in CONYAH	89.9	88.9	71.0	51.1
CONYAH in BZDIOX	68.0	67.3	60.8	59.5
WARPOW in CONYAH	87.6	86.9	71.5	73.0
CONYAH in WARPOW	66.0	59.5	20.3	50.3
MEMTED in CONYAH	97.5	97.5	97.5	92.5
CONYAH in MEMTED	90.9	86.3	54.3	75.8
BZDIOX in WARPOW	69.1	62.2	43.0	31.6
WARPOW in BZDIOX	59.1	59.1	53.3	51.8
MEMTED in WARPOW	87.5	80.0	35.0	60.0
WARPOW in MEMTED	60.6	43.8	3.7	43.8
MEMTED in BZDIOX	85.0	95.0	87.5	85.0
BZDIOX in MEMTED	47.9	35.8	6.2	36.8
VENZAP in VENYUI	90.9	86.4	50.0	86.4
VENYUI in VENZAP	88.9	66.7	22.2	66.7
BENZEN in PRMDIN	81.4	81.4	76.5	74.5
BENZEN in CILWUP	69.6	69.6	68.6	64.7
CILWUP in BENZEN	63.5	63.5	61.4	63.9
CILWUP in PRMDIN	62.2	62.2	56.9	58.1
PRMDIN in BENZEN	59.6	59.6	56.7	57.3
PRMDIN in CILWUP	52.3	52.3	52.3	46.1
PHTHAO in PHALIM	72.6	62.9	45.2	53.2
PHTHAO in INDDON	72.6	72.6	59.7	61.3
PHTHAO in TIPVIZ	66.1	56.5	24.2	50.0
PHTHAO in XUGHUD1	80.7	71.0	38.7	54.8
PHTHAO in XUGHUD2	82.3	71.0	53.2	67.7
PHALIM in PHTHAO	78.4	78.4	66.4	66.4
PHALIM in INDDON	76.0	75.2	72.8	62.4
PHALIM in TIPVIZ	76.8	71.2	25.6	55.2
PHALIM in XUGHUD1	77.6	75.2	41.6	57.6
PHALIM in XUGHUD2	87.2	80.8	72.0	69.6

INDDON in PHTHAO	67.8	67.8	62.1	63.2
INDDON in PHALIM	75.9	74.7	69.0	54.0
INDDON in TIPVIZ	70.1	62.1	27.6	51.7
INDDON in XUGHUD1	75.9	72.4	37.9	51.7
INDDON in XUGHUD2	83.9	78.2	59.8	65.5
TIPVIZ in PHTHAO	100.0	100.0	72.7	81.8
TIPVIZ in PHALIM	90.9	90.9	81.8	81.8
TIPVIZ in INDDON	72.7	72.7	54.6	72.7
TIPVIZ in XUGHUD1	100.0	100.0	90.9	72.7
TIPVIZ in XUGHUD2	90.9	90.9	63.6	90.9
XUGHUD1 in PHTHAO	63.6	61.4	59.1	75.0
XUGHUD1 in PHALIM	77.3	70.5	47.7	68.2
XUGHUD1 in INDDON	70.5	68.2	56.8	75.0
XUGHUD1 in TIPVIZ	72.7	70.5	43.2	72.7
XUGHUD2 in PHTHAO	67.3	63.5	53.9	67.3
XUGHUD2 in PHALIM	67.3	67.3	67.3	57.7
XUGHUD2 in INDDON	59.6	55.8	42.3	53.9
XUGHUD2 in TIPVIZ	53.9	38.5	15.4	44.2
XUGHUD1 in XUGHUD2	86.4	75.0	54.6	56.8
XUGHUD2 in XUGHUD1	82.7	76.9	25.0	55.8
ETHLEN in KEMZIL	35.7	35.9	35.5	29.3
KEMZIL in ETHLEN	62.2	62.2	62.2	56.6

Table. S7: Percetage of low-energy ($\leq$ 7.5 kJ/mol target structures recover (RP) for templating CSP for each template/target molecule pairing for different template selection windows and for quasi-random CSP with sampling equivalent to that used in templating CSP with the corresponding template/target pair and a 25 kJ/mol template selection window

8 Additional Graphs of Recovery Percentage per Family

Figures S4 and S5 show the recovery percentages of low energy-target structures for each template/target pair, separated by family, when using all available templates within the full landscape or within a 15 kJ/mol window respectively. A target structure is defined as recovered if there exists a predicted structure for which 30/30 molecules of 30 molecule clusters of the predicted and known crystal structures can be overlaid to within tolerances of 20% for distances and 20° for angles such that the $RMSD_{30}$ of the overlay is less than or equal to 0.05Å.

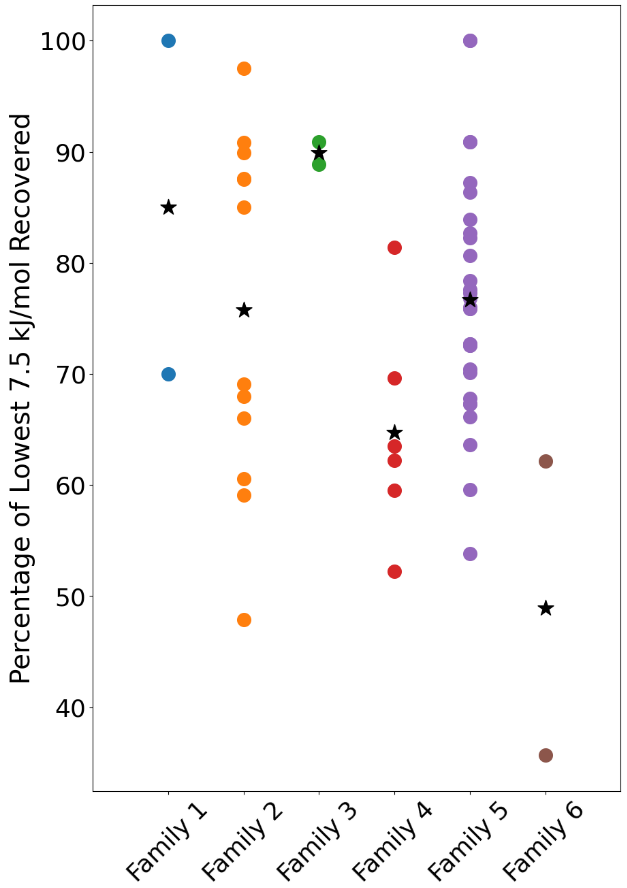

Fig. S4: Plot showing the recovery percentage values for templating CSP runs, using a full landscape template selection window. Results are grouped by the family of similar molecules (See main paper Figure 7) to which the template/target pair belongs.

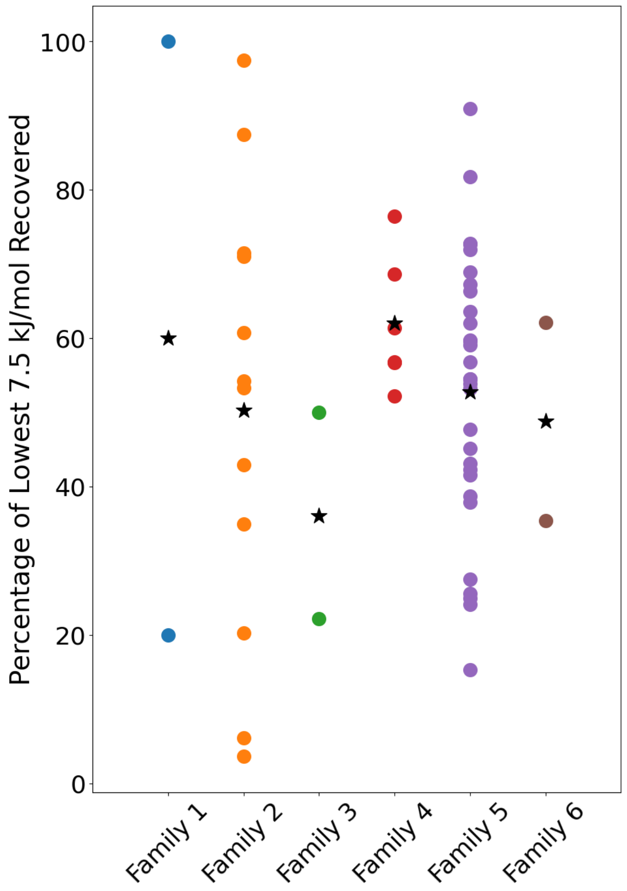

Fig. S5: Plot showing the recovery percentage values for templating CSP runs, using a 15 kJ/mol template selection window. Results are grouped by the family of similar molecules (See main paper Figure 7) to which the template/target pair belongs.

9 Recovery of Low Energy Target Structures with Looser Criterion

We aimed to ensure that the trend, indicated in the main text, of templating CSP outperforming quasi-random CSP was not unfairly biased by the strict criterion used to assess recovery of low-energy target structures. To test this, for a small subset of explored cases (one template/target molecule pair per molecule family), we re-assessed the recovery of low energy target structures using a looser criterion to classify target structures as recovered.

Recall that recovery percentage (RP) is the percentage of unique low energy minima on the target CSP landscape that can be recovered by the CSP method being assessed. For this investigation, we classified, a target structure as recovered if there existed at least one CSP structure for which 30/30 molecules can be overlaid to within tolerances of 20% for distances and 20° for angles such that the $RMSD_{30}$ of the overlay was ≤ 0.3 Å.

Case	Templating RP (%)	QR RP (%)
NTCDA in PTCDA	100.0	100.0
BZDIOX in WARPOW	63.5	45.0
VENYUI in VENZAP	77.8	66.7
BENZEN in PRMDIN	86.3	77.5
PHATHAO in PHALIM	62.9	53.2
ETHLEN in KEMZIL	39.6	33.0

Table. S8: RP values for templating CSP runs, one per molecule family, using 25 kJ/mol template selection window and the respective RP values for a QR CSP run using equivalent sampling. RP values were assessed using a loose criterion of RMSD ≤ 0.3Å to classify a structure as recovered

This recovery of low-energy target structures as assessed using the looser criterion maintained the trend of templating CSP outperforming quasi-random CSP with equivalent sampling (Figure S6), leading to the conclusion that this performance gap represents a real difference between the approaches and was not an artefact of the strict criterion benefiting one method or another.

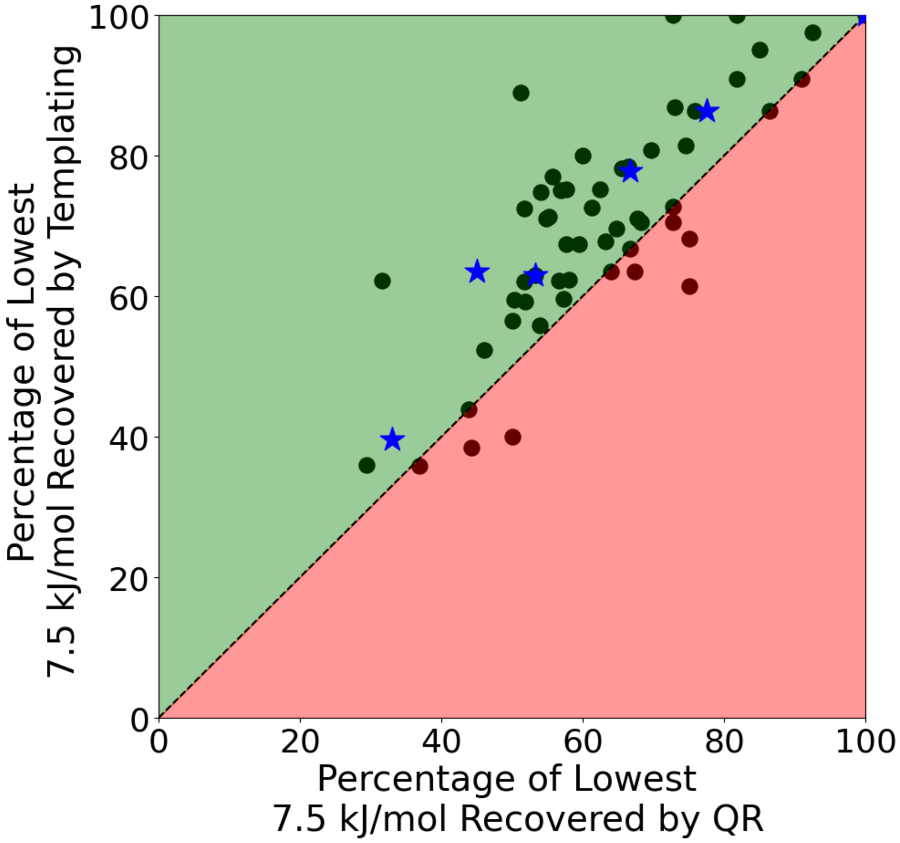

Fig. S6: Scatter plot of the RP values for each templating CSP run using 25 kJ/mol template selection window and the respective RP values for a QR CSP run using equivalent sampling. Points above the line $x = y$ indicate superior performance of the templating approach, with greater height above the line representing greater advantage. Points marked by circles represent the performance when assessed with the strict RMSD $\leq$ 0.05 Å criterion. Points marked by stars represent performance assessed with the looser RMSD $\leq$ 0.3 Å criterion.

10 Minimisation Timings and Performance with Alternative Workflows

We made brief investigation into how altered minimisation workflows would impact upon the average successful lattice-energy minimisation times for structures generated via templating CSP and via quasi-random CSP. We explored 1-step and 2-step minimisation workflows outlined below. In each case the forcefield and charges/multipoles were those used in producing the original target landscapes (See Sec 1 for NTCDA/PTCDA and ref [1] for all other systems.)

1-Step workflow:

1. minimisation with forcefield + multipoles, conducted in DMACRYS [23].

2-Step workflow:

1. minimisation at high pressure (0.1 GPa, forcefield + point charges), conducted in DMACRYS [23]
2. minimisation with forcefield + multipoles, conducted in DMACRYS [23].

Tables S9 and S10 show the average successful geometry optimisation times for example template/target pairings (one pairing per family explored) and the average successful geometry optimisation times for the respective equivalent QR CSP runs, using 1-step and 2-step minimisation workflows respectively. Times indicated are the average, across all successful optimisations, for a single trial structure to complete geometry optimisation. The template selection window used in each case was the 25 kJ/mol selection window.

Case	Avg QR min time (s)	Avg templating min time (s)	Time Reduction (%)
NTCDA in PTCDA	30.71	16.65	45.80
BZDIOX in WARPOW	36.81	15.99	56.57
VENYUI in VENZAP	46.85	22.66	51.65
BENZEN in PRMDIN	22.54	11.68	48.21
PHATHAO in PHALIM	24.66	11.15	54.77
ETHLEN in KEMZIL	10.41	7.32	29.65

Table. S9: Average successful geometry optimisation times, using a 1 step minimisation workflow, for example template/target pairings (one pairing per family explored) and the average successful geometry optimisation times for the respective equivalent QR CSP runs.

Case	Avg QR min time (s)	Avg templating min time (s)	Time Reduction (%)
NTCDA in PTCDA	18.51	13.11	29.16
BZDIOX in WARPOW	17.62	9.94	43.55
VENYUI in VENZAP	17.48	12.11	30.74
BENZEN in PRMDIN	9.31	6.09	34.65
PHATHAO in PHALIM	11.69	7.52	35.71
ETHLEN in KEMZIL	4.55	3.73	18.08

Table. S10: Average successful geometry optimisation times, using a 2 step minimisation workflow, for example template/target pairings (one pairing per family explored) and the average successful geometry optimisation times for the respective equivalent QR CSP runs.

This data may suggest that, for templating CSP, the cost of geometry optimisations could be reduced by adopting the 2-step minimisation workflow. However, it is also important to explore any impact of the minimisation workflow chosen upon performance. We assessed the recovery of low energy target structures for the CSP sets that used the 2-step and 1-step minimisation workflows (Tables S11 and S12 respectively).

Case	Original Templating RP (%)	Original Small QR RP (%)	2-Step Templating RP (%)	2-Step Small QR RP (%)
NTCDA in PTCDA	100.0	100.0	100.0	100.0
BZDIOX in WARPOW	62.2	31.6	46.3	31.3
VENYUI in VENZAP	66.7	66.7	77.8	66.7
BENZEN in PRMDIN	81.4	74.5	84.3	80.4
PHATHAO in PHALIM	62.9	53.2	71.0	53.2
ETHLEN in KEMZIL	35.9	29.3	35.4	32.4

Table. S11: Percentage of Low-energy target structures ($\leq$ 7.5 kJ/mol) recovered by templating CSP using a 25 kJ/mol template selection window, or quasi random CSP using equivalent sampling. Results are shown for test cases using the 'Original' (3-step) minimisation workflow and for cases using a 2-step minimisation workflow

Case	Original Templating RP (%)	Original Small QR RP (%)	1-Step Templating RP (%)	1-Step Small QR RP (%)
NTCDA in PTCDA	100.0	100.0	100.0	100.0
BZDIOX in WARPOW	62.2	31.6	47.6	32.2
VENYUI in VENZAP	66.7	66.7	66.7	88.9
BENZEN in PRMDIN	81.4	74.5	82.4	70.6
PHATHAO in PHALIM	62.9	53.2	66.1	56.5
ETHLEN in KEMZIL	35.9	29.3	33.9	30.4

Table. S12: Percentage of Low-energy target structures ($\leq$ 7.5 kJ/mol) recovered by templating CSP using a 25 kJ/mol template selection window, or quasi random CSP using equivalent sampling. Results are shown for test cases using the 'Original' (3-step) minimisation workflow and for cases using a 1-step minimisation workflow

This data showed a variable impact of altering the minimisation workflow upon the performance of CSP. Therefore, at this stage, we do not intent to alter the existing workflow, which is established and has been shown to produce effective results [2, 24, 25].

11 Factors Influencing Implementation Time

At this juncture, due to the nature of the templating CSP as a proof of concept, for which a fully optimised workflow and program has not been developed, we do not compare full implementation costs against the well-developed quasi-random CSP process (mol-CSpy) [2, 26] used.

However, here we outline a few considerations that are likely to impact the costs of implementing the full templating/quasi-random CSP workflows, and discuss changes to the templating CSP process to help improve overall implementation costs.

One key factor influencing the costs of the CSP methods is the cost of producing trial structures. This process can be achieved with reasonable speed for both approaches for the systems explored. However, it is important to note that the number of analogues per template - and so the cost of constructing the set of analogues - will increase significantly with both increasing Z' and increasing instances/symmetry of the shared substructure. This is due to the need to optimise the substructure overlay for large numbers of possibilities (valid atom-atom mappings to attempt to overlay). This cost of maximising overlay is not shared by quasi-random approaches. As the work develops beyond the proof of concept, consideration should be given to identifying fast algorithms/implementations for this processes.

Currently, construction of initial analogues is performed in serial, whereas construction of quasi-random trial structures is parallelised in the usual implementation of the mol-CSPy [26] code used. It should not be prohibitively difficult to parallelise the construction of analogues for templating CSP. Whilst parallelisation will not reduce the true computational cost of templating CSP, it will reduce the real-time taken, which is crucial for fast CSP applications such as concept testing or integration with genetic algorithms[27].

Another key factor, currently presenting a bottleneck for templating CSP is the cost of de-duplicating the set of analogues prior to lattice-energy minimisation. This can be costly, as the automated nature of constructing analogues for each valid substructure overlay often leads to the construction of duplicate analogues, for which the substructure overlays implemented differ, but the resulting crystal structures do not. Possibilities for reducing this bottleneck include steps to prevent the formation of duplicate analogues or the use of high-speed de-duplication approaches such as the use of structural invariants [28].

12 Landscape Correlations

Table S13 shows the correlation coefficients and p-values for the Kendall-rank correlation between the energetic ranking of template crystal structures and the energetic rankings of analogues formed from them. For each template/target pair we compared the rankings of a full landscape of templates and their respective analogues. Each template was mapped to its lowest energy analogue, templates not corresponding to any analogue or vice versa were disregarded.

Prior to calculation of rank correlation, the analogue sets de-duplicated using simulated pXRD pattern comparisons as outlined in the main text.

Case	Correlation	Significance
NTCDA in PTCDA	0.39	1.15E-30
PTCDA in NTCDA	0.22	4.73E-10
MEMTED in BZDIOX	0.31	1.34E-84
BZDIOX in MEMTED	0.09	0.00
BZDIOX in CONYAH	0.41	4.50E-173
CONYAH in BZDIOX	0.39	3.27E-159
WARPOW in CONYAH	0.27	2.69E-104
CONYAH in WARPOW	0.12	8.90E-13
MEMTED in CONYAH	0.33	3.71E-164
CONYAH in MEMTED	0.22	1.74E-38
BZDIOX in WARPOW	0.25	1.28E-34
WARPOW in BZDIOX	0.35	6.07E-99
MEMTED in WARPOW	0.02	0.41
WARPOW in MEMTED	0.01	0.64
VENZAP in VENYUI	0.23	2.13E-12
VENYUI in VENZAP	0.19	1.38E-08
BENZEN in PRMDIN	0.36	1.63E-19
BENZEN in CILWUP	0.39	4.89E-17
CILWUP in BENZEN	0.32	7.71E-15
CILWUP in PRMDIN	0.32	4.14E-21
PRMDIN in BENZEN	0.30	3.28E-16
PRMDIN in CILWUP	0.36	8.79E-27
PHATHAO in PHALIM	0.18	1.27E-12

PHATHAO in INDDON	0.36	1.62E-54
PHATHAO in TIPVIZ	0.11	8.06E-05
PHATHAO in XUGHUD1	0.11	7.86E-05
PHATHAO in XUGHUD2	0.26	1.66E-47
PHALIM in PHATHAO	0.20	2.21E-19
PHALIM in INDDON	0.42	8.18E-80
PHALIM in TIPVIZ	0.22	1.54E-14
PHALIM in XUGHUD1	0.25	3.27E-21
PHALIM in XUGHUD2	0.41	8.32E-138
INDDON in PHATHAO	0.34	8.91E-50
INDDON in PHALIM	0.46	1.65E-86
INDDON in TIPVIZ	0.19	1.23E-10
INDDON in XUGHUD1	0.18	2.38E-11
INDDON in XUGHUD2	0.31	4.73E-79
TIPVIZ in PHATHAO	0.18	1.72E-16
TIPVIZ in PHALIM	0.28	2.17E-33
TIPVIZ in INDDON	0.24	5.94E-30
TIPVIZ in XUGHUD1	0.22	1.80E-19
TIPVIZ in XUGHUD2	0.30	1.24E-09
XUGHUD1 in PHATHAO	0.21	1.26E-22
XUGHUD1 in PHALIM	0.24	3.50E-25
XUGHUD1 in INDDON	0.23	1.34E-28
XUGHUD1 in TIPVIZ	0.17	2.57E-11
XUGHUD2 in PHATHAO	0.19	1.81E-20
XUGHUD2 in PHALIM	0.29	4.89E-41
XUGHUD2 in INDDON	0.27	3.73E-42
XUGHUD2 in TIPVIZ	0.18	9.28E-13
XUGHUD1 in XUGHUD2	0.30	1.32E-86
XUGHUD2 in XUGHUD1	0.05	0.06
ETHLEN in KEMZIL	0.30	5.19E-09
KEMZIL in ETHLEN	0.31	9.89E-12

Table. S13: Correlation coefficients and p-values for the Kendall-rank correlation between the energetic ranking of template crystal structures and the energetic rankings of lowest energy analogues formed from them

13 Distribution of Matches

Our main text findings of significant correlation between CSP landscapes of similar molecules suggested that low energy templates may be more likely to lead to low energy analogues. If this is the case, the success of templating - relative to the number of minimisations performed - should increase when beginning templating from a low energy window on the original landscape. That is, that the efficiency of the approach should increase when using templates sourced from a low energy window rather than from across a full landscape. We quantify this by taking a statistic we call the distribution of matches (DOM), which is given by the sampling efficiency (SE) of templating CSP using templates selected from a low-energy window relative to templating CSP using all available templates.:

$$\mathrm{SE} = \frac{\text{No. unique low energy traditional minima recovered}}{\text{No. unique starting analogues}} \tag{1}$$

$$\mathrm{DOM} = \frac{\text{SE using template selection}}{\text{SE using all templates}} \tag{2}$$

We calculated this measure for all template-target pairs and template selection approaches investigated. (A summary is provided in Table S14 and a full table of DOM values for each template/target pair is provided in Table S15

Template Selection Window	Mean DOM	Maximum DOM
25 kJ/mol	1.7	9.2
15 kJ/mol	3.6	21.0

Table. S14: Mean and Maximum distribution of matches (DOM) values, across all template target-pairs investigated, when using 25 kJ/mol and 15 kJ/mol template selection windows

Case	DOM 25 kJ/mol	DOM 15 kJ/mol
NTCDA in PTCDA	9.2	21.0
PTCDA in NTCDA	3.0	10.5
BZDIOX in CONYAH	1.3	4.0
CONYAH in BZDIOX	1.2	2.2

WARPOW in CONYAH	1.3	4.1
CONYAH in WARPOW	1.2	1.0
MEMTED in CONYAH	1.3	5.3
CONYAH in MEMTED	1.7	5.5
BZDIOX in WARPOW	1.2	2.1
WARPOW in BZDIOX	1.2	2.3
MEMTED in WARPOW	1.2	1.3
WARPOW in MEMTED	1.3	0.5
MEMTED in BZDIOX	1.3	2.4
BZDIOX in MEMTED	1.4	1.1
VENZAP in VENYUI	1.6	6.0
VENYUI in VENZAP	1.1	1.5
BENZEN in PRMDIN	1.0	1.5
BENZEN in CILWUP	1.0	1.3
CILWUP in BENZEN	1.0	1.3
CILWUP in PRMDIN	1.0	1.5
PRMDIN in BENZEN	1.0	1.3
PRMDIN in CILWUP	1.0	1.3
PHTHAO in PHALIM	1.2	1.9
PHTHAO in INDDON	1.3	2.8
PHTHAO in TIPVIZ	1.5	4.0
PHTHAO in XUGHUD1	1.5	3.0
PHTHAO in XUGHUD2	3.0	7.4
PHALIM in PHTHAO	1.2	2.1
PHALIM in INDDON	1.3	3.3
PHALIM in TIPVIZ	1.6	3.7
PHALIM in XUGHUD1	1.7	3.3
PHALIM in XUGHUD2	3.2	9.5
INDDON in PHTHAO	1.2	2.2
INDDON in PHALIM	1.3	2.7
INDDON in TIPVIZ	1.5	4.4
INDDON in XUGHUD1	1.7	3.1
INDDON in XUGHUD2	3.3	8.2

TIPVIZ in PHTHAO	1.2	1.8
TIPVIZ in PHALIM	1.4	2.7
TIPVIZ in INDDON	1.3	2.6
TIPVIZ in XUGHUD1	1.7	5.7
TIPVIZ in XUGHUD2	3.5	8.0
XUGHUD1 in PHTHAO	1.2	2.3
XUGHUD1 in PHALIM	1.2	1.8
XUGHUD1 in INDDON	1.3	2.7
XUGHUD1 in TIPVIZ	1.7	6.3
XUGHUD2 in PHTHAO	1.2	2.0
XUGHUD2 in PHALIM	1.3	2.9
XUGHUD2 in INDDON	1.2	2.4
XUGHUD2 in TIPVIZ	1.2	3.2
XUGHUD1 in XUGHUD2	3.0	7.3
XUGHUD2 in XUGHUD1	1.6	1.9
ETHLEN in KEMZIL	1.0	1.0
KEMZIL in ETHLEN	1.0	1.0

Table. S15: Distribution of matches (DOM) values for each templating-target molecule pair, calculated for the 25 kJ/mol and 15 kJ/mol template selection windows.

These results corroborated the proposal that low energy templates are more likely to lead to unique low energy analogues. In almost all cases, the DOM value was found to be greater than or equal to one, in some instances being far greater than one, demonstrating a notable improvement in efficiency when using more restrictive template selection windows. The consistency of this trend, alongside the moderate rank correlation of template and analogue landscapes leads us to conclude that there is a meaningful relationship between the CSP landscapes of similar molecules.

14 Landscape Correlation vs Performance

Template Selection Window	Correlation	Significance
Full Landscape	-0.01	0.90
25 kJ/mol	0.06	0.50
15 kJ/mol	0.30	0.00

Table. S16: Table showing the kendall rank correlation, and associated p-value, between the list of rank-correlations for each template-target molecule pair, across the full landscape of templates (See S13) and the corresponding list of 'performance values' for templating CSP with different template selection windows. The performance measure used is the percentage of low-energy ($\leq$ 7.5 kJ/mol target structures recovered (See S7)

References

(1) Taylor, C.; Butler, P.; Day, G. *Faraday Discuss.* **2025**, *256*, 434–458.

(2) Case, D. H.; Campbell, J. E.; Bygrave, P. J.; Day, G. M. *J. Chem. Theory Comput.* **2016**, *12*, 910–924.

(3) Becke, A. D. *J. Chem. Phys.* **1993**, *98*, 5648–5652.

(4) Stephens, P. J.; Devlin, F. J.; Chabalowski, C. F.; Frisch, M. J. *J. Chem. Phys.* **1994**, *98*, 11623–11627.

(5) Lee, C.; Yang, W.; Parr, R. G. *Phys. Rev. B* **1988**, *37*, 785–789.

(6) Grimme, S.; Ehrlich, S.; Goerigk, L. *J. Comput. Chem.* **2011**, *32*, 1456–1465.

(7) Krishnan, R.; Binkley, J. S.; Seeger, R.; Pople, J. A. *J. Chem. Phys.* **1980**, *72*, 650–654.

(8) Frisch, M. J.; Trucks, G. W.; Schlegel, H. B.; Scuseria, G. E.; Robb, M. A.; Cheeseman, J. R.; Scalmani, G.; Barone, V.; Mennucci, B.; Petersson, G. A.; Nakatsuji, H.; Caricato, M.; Li, X.; Hratchian, H. P.; Izmaylov, A. F.; Bloino, J.; Zheng, G.; Sonnenberg, J. L.; Hada, M.; Ehara, M.; Toyota, K.; Fukuda, R.; Hasegawa, J.; Ishida, M.; Nakajima, T.; Honda, Y.; Kitao, O.; Nakai, H.; Vreven, T.; Montgomery, J. A.; Peralta, J. E.; Ogliaro, F.; Bearpark, M.; Heyd, J. J.; Brothers, E.; Kudin, K. N.; Staroverov, V. N.; Kobayashi, R.; Normand, J.; Raghavachari, K.; Rendell, A.; Burant, J. C.; Iyengar, S. S.; Tomasi, J.; Cossi, M.; Rega, N.; Millam, J. M.; Klene, M.; Knox, J. E.; Cross, J. B.; Bakken, V.; Adamo, C.; Jaramillo, J.; Gomperts, R.; Stratmann, R. E.; Yazyev, O.; Austin, A. J.; Cammi, R.; Pomelli, C.; Ochterski, J. W.; Martin, R. L.; Morokuma, K.; Zakrzewski, V. G.; Voth, G. A.; Salvador, P.; Dannenberg, J. J.; Dapprich, S.; Daniels, A. D.; Farkas, Ö; Foresman, J. B.; Ortiz, J. V.; Cioslowski, J.; Fox, D. J. Gaussian 09 Revision A.2, 2009.

(9) Cox, S.; Hsu, L.-Y.; Williams, D. *Acta Crystallographica* **1981**, *37*, 293–301.

(10) Perdew, J. P.; Ernzerhof, M.; Burke, K. *J. Chem. Phys.* **1996**, *105*, 9982–9985.

(11) Adamo, C.; Barone, V. *J. Chem. Phys.* **1999**, *110*, 6158–6170.

(12) Ditchfield, R.; Hehre, W. J.; Pople, J. A. *J. Chem. Phys.* **1971**, *54*, 724–728.

(13) Hariharan, P. C.; Pople, J. A. *Theor. Chim. Acta* **1973**, *28*, 213–222.

(14) Hehre, W. J.; Ditchfield, R.; Pople, J. A. *J. Chem. Phys.* **1972**, *56*, 2257–2261.

(15) Groom, C. R.; Bruno, I. J.; Lightfoot, M. P.; Ward, S. C. *Acta Crystallogr., Sect. B* **2016**, *72*, 171–179.

(16) Chisholm, J.; Motherwell, S. *Journal* **2005**, *38*, 228–231.

(17) Sykes, R. A.; Johnson, N. T.; Kingsbury, C. J.; Harter, J.; Maloney, A. G. P.; Sugden, I. J.; Ward, S. C.; Bruno, I. J.; Adcock, S. A.; Wood, P. A.; McCabe, P.; Moldovan, A. A.; Atkinson, F.; Giangreco, I.; Cole, J. C. *Journal of Applied Crystallography* **2024**, *57*, 1235–1250.

(18) Born, L; Heywang, G *Zeitschrift fÃijr Kristallographie* **1990**, *190*, 147–152.

(19) Tojo, Î.; Mizuguchi, J. *Zeitschrift fÃijr Kristallographie - New Crystal Structures* **2002**, *217*, 253–254.

(20) Cuthill, E.; McKee, J. In *Proceedings of the 1969 24th National Conference*, Association for Computing Machinery: New York, NY, USA, 1969, 157âĂŞ172.

(21) Virtanen, P.; Gommers, R.; Oliphant, T. E.; Haberland, M.; Reddy, T.; Cournapeau, D.; Burovski, E.; Peterson, P.; Weckesser, W.; Bright, J.; van der Walt, S. J.; Brett, M.; Wilson, J.; Millman, K. J.; Mayorov, N.; Nelson, A. R. J.; Jones, E.; Kern, R.; Larson, E.; Carey, C. J.; Polat, İ.; Feng, Y.; Moore, E. W.; VanderPlas, J.; Laxalde, D.; Perktold, J.; Cimrman, R.; Henriksen, I.; Quintero, E. A.; Harris, C. R.; Archibald, A. M.; Ribeiro, A. H.; Pedregosa, F.; van Mulbregt, P.; SciPy 1.0 Contributors *Nature Methods* **2020**, *17*, 261–272.

(22) J.Martin, PhD Thesis, Machine Learning Methods for Analysis of Organic Molecular Crystal Structure Prediction Landscapes, School of Chemistry and Chemical Engineering, University of Southampton, 2025.

(23) Price, S. L.; Leslie, M.; Welch, G. W. A.; Habgood, M.; Price, L. S.; Karamertzanis, P. G.; Day, G. M. *Phys. Chem. Chem. Phys.* **2010**, *12*, 8478–8490.

(24) Hunnisett, L. M. et al. *Acta Crystallographica Section B* **2024**, *80*, 548–574.

(25) Nyman, J.; Pundyke, O. S.; Day, G. M. *Phys. Chem. Chem. Phys.* **2016**, *18*, 15828–15837.

(26) mol-CSPy GitLab,`https://gitlab.com/mol-cspy/mol-cspy`,(Accessed: 2025-03-18).

(27) Johal, J; Day, G. M. *Nat Commun* **2025**, *16*, 10540.

(28) Widdowson, D. E.; Kurlin, V. A. *Crystal Growth & Design* **2024**, *24*, 5627–5636.
